# Single-cell sequencing reveals Hippo signaling as a driver of fibrosis in hidradenitis suppurativa

**DOI:** 10.1172/JCI169225

**Published:** 2024-02-01

**Authors:** Kelsey R. van Straalen, Feiyang Ma, Pei-Suen Tsou, Olesya Plazyo, Mehrnaz Gharaee-Kermani, Marta Calbet, Xianying Xing, Mrinal K. Sarkar, Ranjitha Uppala, Paul W. Harms, Rachael Wasikowski, Lina Nahlawi, Mio Nakamura, Milad Eshaq, Cong Wang, Craig Dobry, Jeffrey H. Kozlow, Jill Cherry-Bukowiec, William D. Brodie, Kerstin Wolk, Özge Uluçkan, Megan N. Mattichak, Matteo Pellegrini, Robert L. Modlin, Emanual Maverakis, Robert Sabat, J. Michelle Kahlenberg, Allison C. Billi, Lam C. Tsoi, Johann E. Gudjonsson

**Affiliations:** 1 Department of Dermatology and; 2 Division of Rheumatology, Department of Internal Medicine, University of Michigan Medical School, Ann Arbor, Michigan, USA.; 3 Almirall SA, R&D Center, Sant Feliu de Llobregat, Barcelona, Spain.; 4 Department of Pathology, University of Michigan Medical School, Ann Arbor, Michigan, USA.; 5 Laboratory for Experimental Immunodermatology, Department of Dermatology, Erasmus University Medical Center, Rotterdam, Netherlands.; 6 Division of Plastic Surgery, Department of Surgery, and; 7 Section of General Surgery, Department of Surgery, University of Michigan Medical School, Ann Arbor, Michigan, USA.; 8 Interdisciplinary group Molecular Immunopathology, Dermatology/Medical Immunology, Charité – Universitätsmedizin Berlin, Berlin, Germany.; 9 Department of Molecular, Cell, and Developmental Biology and; 10 Department of Dermatology, UCLA, Los Angeles, California, USA.; 11 Department of Dermatology, University of California, Sacramento, California, USA.

**Keywords:** Dermatology, Inflammation, Cytokines, Fibrosis, Skin

## Abstract

Hidradenitis suppurativa (HS) is a chronic inflammatory disease characterized by abscesses, nodules, dissecting/draining tunnels, and extensive fibrosis. Here, we integrate single-cell RNA sequencing, spatial transcriptomics, and immunostaining to provide an unprecedented view of the pathogenesis of chronic HS, characterizing the main cellular players and defining their interactions. We found a striking layering of the chronic HS infiltrate and identified the contribution of 2 fibroblast subtypes (*SFRP4^+^* and *CXCL13^+^*) in orchestrating this compartmentalized immune response. We further demonstrated the central role of the Hippo pathway in promoting extensive fibrosis in HS and provided preclinical evidence that the profibrotic fibroblast response in HS can be modulated through inhibition of this pathway. These data provide insights into key aspects of HS pathogenesis with broad therapeutic implications.

## Abstract 2

<p>Chronic hidradenitis suppurativa is orchestrated by pro-inflammatory fibroblast subsets that shape the immune infiltrate and promote aberrant, Hippo pathway-dependent fibrosis.</p>

## Graphical abstract

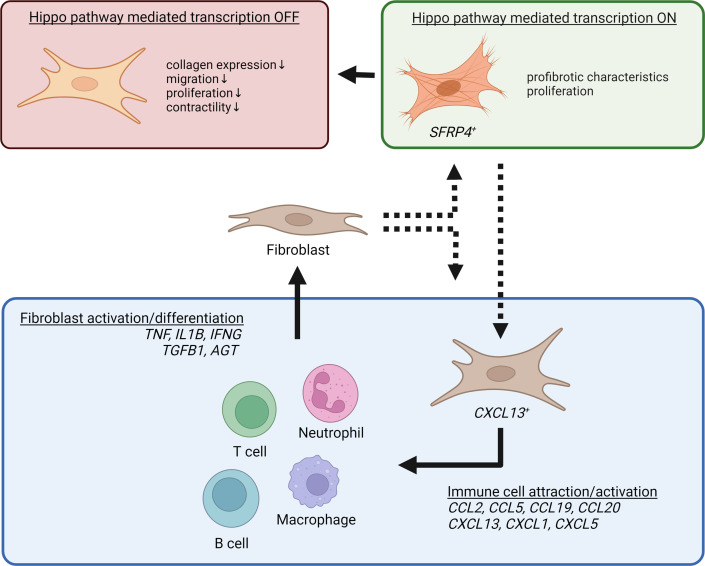

## Introduction

Hidradenitis suppurativa (HS) is a chronic inflammatory disease of the skin that affects 1% of the general population (1). The disease is characterized by acute, recurrent inflammatory nodules and painful abscesses originating from the hair follicles, typically arising in the axillae and groin (2, 3). Later stages of HS are marked by chronic, persistent inflammation accompanied by dermal tunnel (sinus tract) formation and extensive fibrosis (2, 3).

While the exact pathogenesis of HS remains unknown, genetic predisposition and environmental factors such as cigarette smoking and obesity may contribute to the disease (2, 4–6). The primary pathogenic event is thought to be infundibular hyperplasia arising from an intrinsic keratinocyte defect (2, 7). Subsequent cyst formation and rupture induce acute inflammation, characterized by a mixed immune infiltrate of neutrophils, macrophages, dendritic cells, and T and B cells and increased expression of a battery of proinflammatory cytokines including IL-1β, IL-17, and TNF-α (2, 8). Chronic lesions are thought to develop upon repeated rupture or failure to clear the inflammatory follicle contents. These lesions show a shift in immune cell composition marked by more prominent B cell and plasma cell components (9). Dermal tunnels are a hallmark of these chronic lesions, yet the processes that lead to their development remain unknown. Fibrosis is a prominent clinical feature of long-standing HS (2). The relationship of fibrosis to the HS inflammatory response and the mechanisms involved have not been characterized but are of high importance, as fibrosis may interfere with drug penetration and impact overall treatment response (2).

Treatment options for this debilitating disease remain limited, with the only FDA-approved therapy (adalimumab) achieving clinical response in only 40% to 60% of patients (10). Response rates among newer, repurposed, biological therapies in large clinical trials have so far failed to exceed this (10, 11). A major barrier to the identification of treatment targets and successful clinical translation is our lack of understanding of the interplay between the different cell types — both immune and stromal — in the HS microenvironment. The contribution of stromal cells, while clearly implicated by clinical symptoms of tissue destruction and fibrosis, has only been explored to a limited extent (12, 13).

In this paper, we use single-cell RNA sequencing (scRNA-Seq) and spatial transcriptomics to define the cellular composition and spatial architecture of the infiltrate in chronic HS lesions. Our results provide an unprecedented view of HS pathology, demonstrating how stromal-immune cell interactions contribute to the inflammatory network at the site of disease and identifying a pathway implicated in HS fibrosis that may serve as a potential target for future therapeutic interventions.

## Results

### Chronic HS lesions show altered cell composition and complex layered architecture.

**Figure 1:**
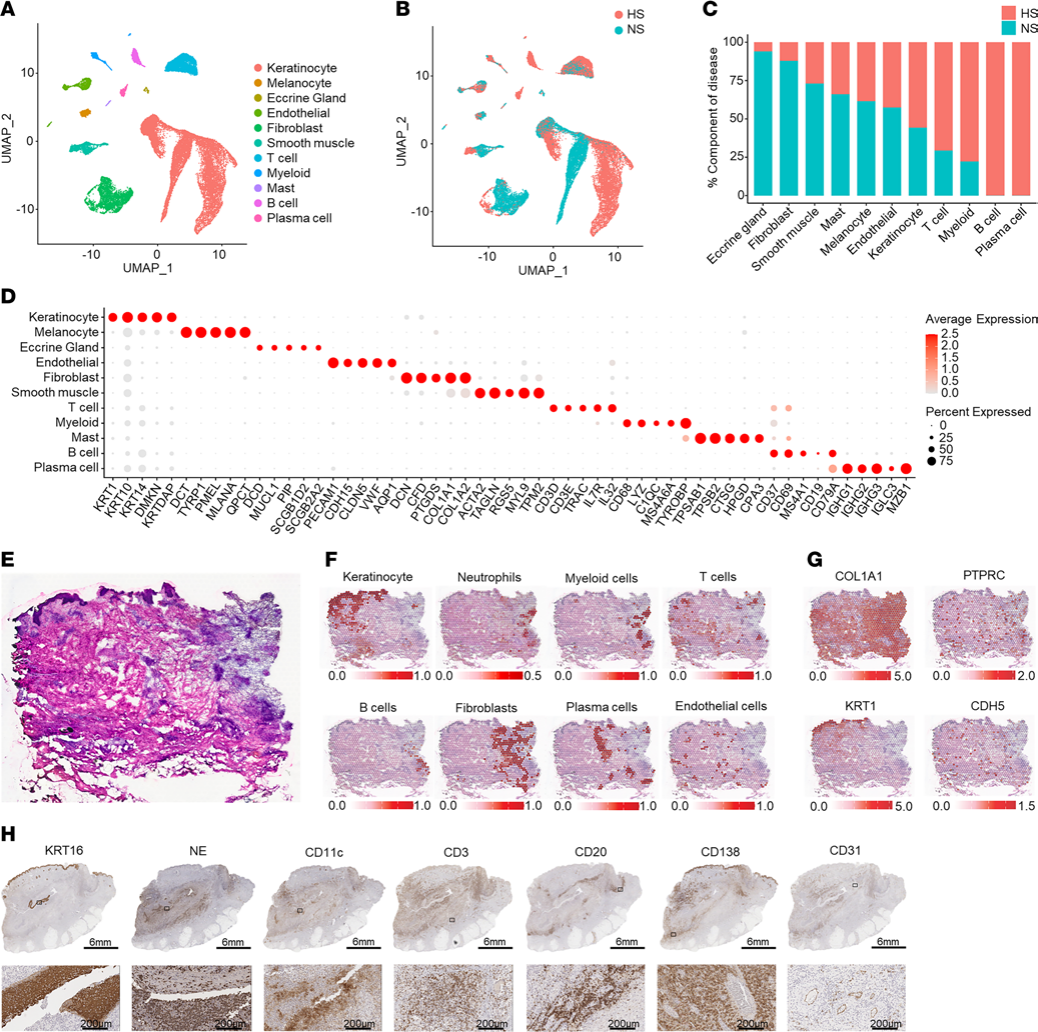
Cell types observed in HS lesional skin and their spatial locations. (**A**) UMAP plot showing 31,746 cells colored by cell type. (**B**) UMAP plot showing the cells colored by disease condition. HS, hidradenitis suppurativa; NS, normal skin from healthy controls. (**C**) Bar chart showing the cell types as percentage component of disease. (**D**) Dot plot showing 5 representative marker genes for each cell type. The color scale represents the scaled expression average of each gene. The size of the dot represents the percentage of cells expressing each gene. (**E**) H&E staining of the biopsy used for spatial transcriptomics. (**F**) Spatial plot showing localization of KCs, neutrophils, myeloid cells, FBs, B cells, plasma cells, and endothelial cells superimposed on H&E slide. (**G**) Spatial plot showing detection of *COL1A1* (encoding collagen 1A1), *PTPRC* (CD45), *KRT1* (keratin 1), and *CDH5* (cadherin 5) within HS lesional skin. (**H**) IHC showing the localization of proliferative KCs (KRT16), neutrophils (NE, neutrophil elastase), T cells (CD3), B cells (CD20), plasma cells (CD138), dendritic cells (CD11c), and endothelial cells (CD31) in HS lesional skin (patient HS1). Scale bars: top, 6 mm; bottom, 200 μm.

To understand the cell composition of healthy and chronic HS lesional skin, we performed scRNA-Seq on cells isolated from chronic lesional skin of 5 HS patients and 8 healthy donors (normal skin [NS]). We collected 31,716 cells and identified 21 clusters that we annotated as 11 distinct primary cell types: keratinocytes (KCs), melanocytes, eccrine gland cells, endothelial cells, fibroblasts (FBs), smooth muscle cells, T cells, myeloid cells, mast cells, B cells, and plasma cells (Figure 1A and Supplemental Figure 1A; supplemental material available online with this article; https://doi.org/10.1172/JCI169225DS1). Interestingly, for all major stromal cell types, including KCs and FBs, uniform manifold approximation and projection (UMAP) showed distinct separation between HS and NS cells, suggesting fundamental transcriptomic changes in the HS-associated cell types (Figure 1, A and B). Analysis of the disease composition for each cell type revealed an increased proportion of KCs and immune cells, particularly T cells, B cells, plasma cells, and myeloid cells, in HS (Figure 1C). This high number of KCs and massive immune cell infiltration, obtained from biopsies in chronic inflammatory lesions, resulted in a relative decreased proportion of eccrine gland cells, FBs, and smooth muscle cells. Figure 1D shows the expression of relevant marker genes for each cell type. These results indicate that chronic HS is characterized by both the accumulation of an abnormal immune infiltrate and a marked transcriptomic shift in all major stromal cell types.

We performed spatial transcriptomics on 4 samples to elucidate the spatial organization of the identified cell types within chronic HS lesional skin. Subsequently, we deconvoluted the RNA expression in each spot with the scRNA-Seq gene expression of the major cell types to identify the cell type composition in each capture spot (Supplemental Figure 1B). As expected, KCs were primarily detected in the epidermis. Interestingly, a layered architecture was seen in chronic HS lesions. Myeloid cells were localized primarily within a large focus of dense inflammation, where T cells are dispersed throughout the infiltrate (Figure 1, E and F, and Supplemental Figure 1C). B cells were found in clusters at the edge of the infiltrate (Figure 1, E and F). The inflammatory infiltrate was demarcated by a layer of FBs, with plasma cells found primarily outside of the FB zone (Figure 1F). Figure 1G demonstrates the localization of *COL1A1*, *PTPRC* (CD45), *KRT1*, and *CDH5* (vascular endothelial cadherin). Immunohistochemistry (IHC) corroborated this layered arrangement of infiltrating immune cells and stromal cells centering around a ruptured tunnel or abscess (Figure 1H).

**Figure 2:**
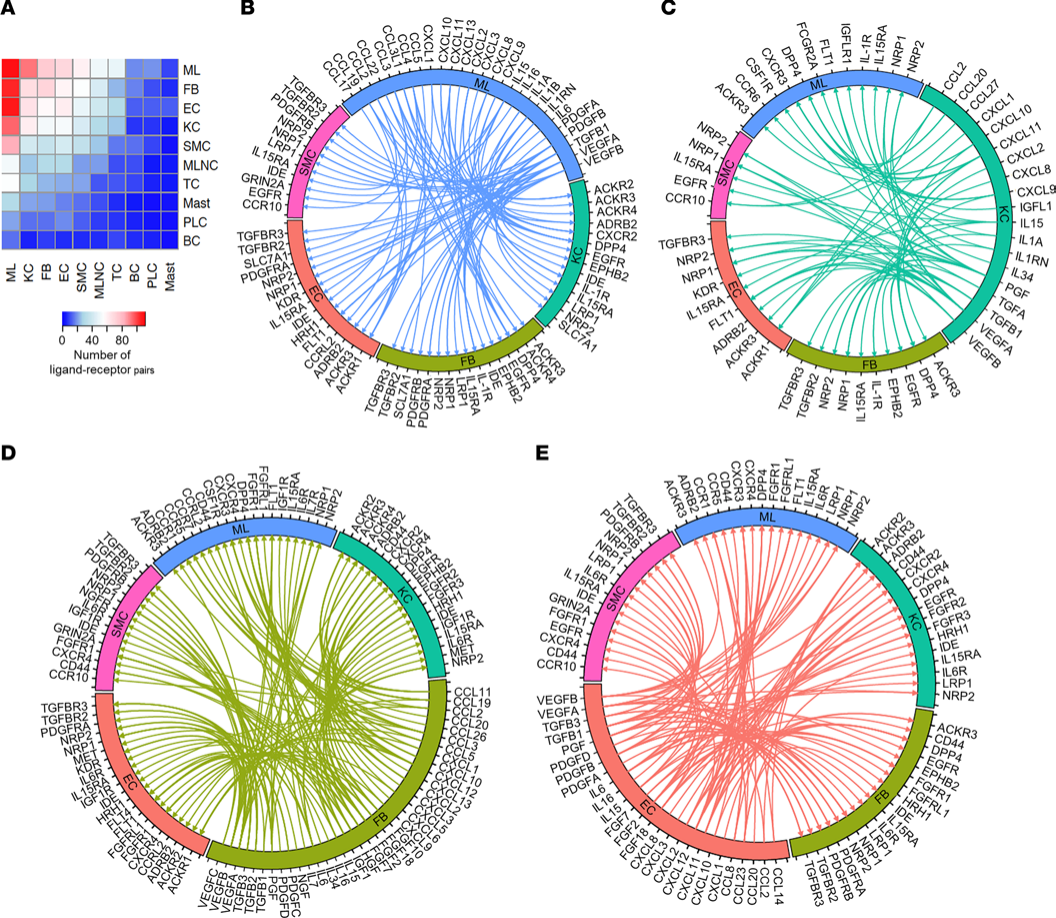
Ligand-receptor interactions between cell types. (**A**) Heatmap showing the number of ligand-receptor pairs with a higher score in HS compared with NS among the cell types. Rows, cell type expressing ligand; columns, cell type expressing receptor. ML, myeloid cells; FB, fibroblasts; EC, endothelial cells; KC, keratinocytes; SMC, smooth muscle cells; MLNC, melanocytes; TC, T cells; Mast, mast cells; PLC, plasma cells; BC, B cells. (**B**–**E**) Circos plots showing cytokine and growth factor ligand-receptor interactions with higher scores in HS compared with NS in which ligands are expressed by myeloid cells (**B**), keratinocytes (**C**), FBs (**D**), and endothelial cells (**E**) with receptors expressed by other cell types.

To analyze the differences in cell-cell communication between HS and healthy skin, we performed ligand-receptor analysis using CellPhoneDB and analyzed the ligand-receptor pairs with higher interaction scores in HS than NS. This identified myeloid cells, KCs, FBs, endothelial cells, smooth muscle cells, and to a lesser extent T cells as the major putative cell interactors in lesional HS skin (Figure 2A). Growth factor and cytokine interactions between myeloid cells, KCs, FBs, endothelial cells, and smooth muscle cells are plotted in Figure 2, B–E (Supplemental Figure 2 for the smooth muscle cells). Myeloid cells showed expression of several growth factors (*VEGFA*, *VEGFB*, *PDGFB*, and *PDGFA*), which link to their respective receptors on KCs, FBs, endothelial cells, and smooth muscle cells, potentially stimulating the proliferation of these cells (Figure 2B). KCs expressed several chemokines (*CXCL9*, *CXCL10*, *CXCL11*) and cytokines (*IL1*, *IL15*) capable of interacting with their respective receptors on myeloid cells and FBs (Figure 2C). FBs expressed a plethora of chemokines (e.g., *CCL19*, *CCL20*, *CXCL2*, *CXCL12*) that bind to receptors on myeloid cells, suggesting an important role for FBs in recruiting immune cells to the HS infiltrate (Figure 2D). Both endothelial cells and smooth muscle cells produced diverse chemokines and growth factors interacting with their respective receptors on KCs, FBs, and myeloid cells (Figure 2E and Supplemental Figure 2). Moreover, multiple chemokines produced by these cell types were predicted to be scavenged from the microenvironment by KCs and FBs through interaction with atypical chemokine receptors *ACKR2*, *ACKR3*, and *ACKR4* (14, 15).

### cDC2B cells and macrophages can promote neutrophil activation and degranulation in HS skin.

**Figure 3:**
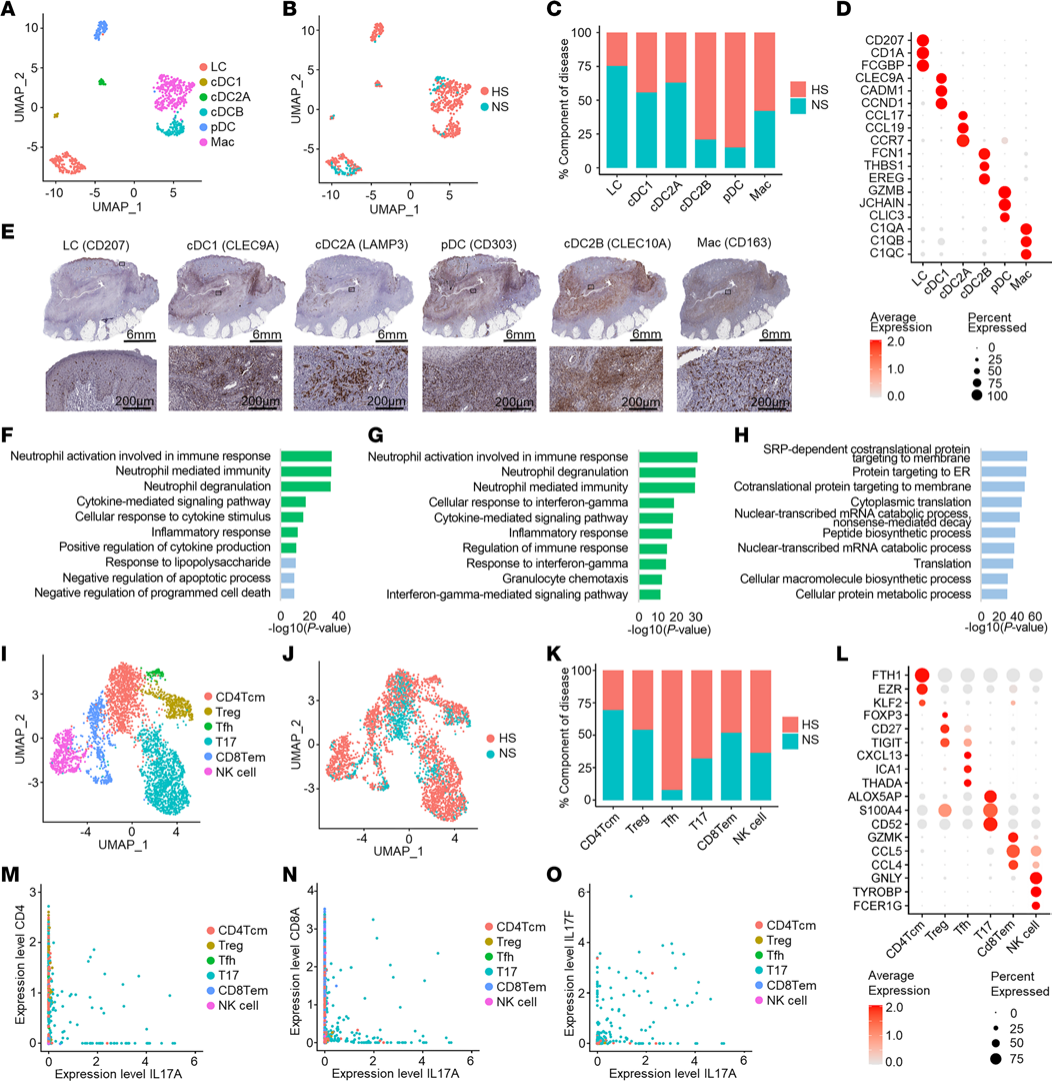
Identification of myeloid cell and T cell subsets in HS lesional skin. (**A**) UMAP showing 689 myeloid cells colored by subtype. (**B**) UMAP showing the cells colored by disease condition. (**C**) Bar chart showing the subtypes as percentage component of disease. (**D**) Dot plot showing representative marker genes for each subtype. Color represents scaled expression; size of the dot represents the percentage of cells expressing the gene. (**E**) IHC showing myeloid cell subtype localization in HS lesional skin (patient HS1). Scale bars: top, 6 mm; bottom, 200 μm. (**F**–**H**) Bar chart showing enriched Gene Ontology biological processes in HS cDC2B cells (**F**), macrophages (**G**), and pDCs (**H**). Green, immune associated; blue, transcription related and other biological processes. (**I**) UMAP showing 3,985 T cells colored by subtype. (**J**) UMAP showing T cells colored by disease condition. (**K**) Bar chart showing the T cell subtypes as percentage component of disease. (**L**) Dot plot showing representative marker genes for T cell subtypes. Color represents scaled expression; size of the dot represents the percentage of cells expressing the gene. (**M** and **N**) Scatterplots showing the correlation between the levels of expression of IL-17A (*x* axis) and CD4 (**M**, ρ = 0.03) or CD8A (**N**, ρ = 0.01). (**O**) Scatterplot showing the correlation between the levels of expression of IL-17A and IL-17F, ρ = 0.44, among T cells.

To examine the heterogeneity in myeloid cells, we subclustered the myeloid cells and annotated 6 subpopulations: Langerhans cells (LCs), classical type 1 dendritic cells (cDC1 cells), classical type 2 dendritic cell subset A (cDC2A cells), classical type 2 dendritic cell subset B (cDC2B cells), plasmacytoid dendritic cells (pDC), and macrophages (Figure 3A). Analysis of the disease composition revealed that pDCs and cDC2B cells were mainly derived from HS lesional skin, which also showed a relative decrease in LCs compared with healthy skin (Figure 3, B and C). Characteristic marker genes for all subpopulations are shown in Figure 3D. IHC revealed distinct spatial localization for many of the myeloid cell subtypes within the layered architecture of chronic HS lesional skin. As expected, LCs were found primarily within the epidermis (Figure 3E). Classical type 1 dendritic cells were found predominantly at the center and edges of the infiltrate, whereas cDC2A cells were found in small clusters within the infiltrate, and the cDC2B cells, pDCs, and macrophages were dispersed throughout the infiltrate (Figure 3E). While our single-cell skin digestion protocol precludes capture of neutrophils, analysis of the enriched biological processes of the cDC2B and macrophage subtypes revealed their extensive involvement in neutrophil activation and degranulation (Figure 3, F and G). pDCs were found to be highly transcriptionally active and show upregulation of general protein translation pathways (Figure 3H).

### IL-17^+^ T cells contribute to the production of both IL17A and IL17F in HS lesional skin.

To assess for dysregulation of T cell subsets in HS, we aimed to characterize the T cell subtypes found in our data. We identified 6 T/NK cell subtypes in HS lesional and healthy skin: CD4^+^ central memory T cells (CD4Tcm), Tregs, T follicular helper cells (Tfh), IL-17^+^ T cells (T17), CD8^+^ effector memory T cells (CD8Tem), and NK cells (Figure 3I). Analysis of the disease composition revealed an increased proportion of Tfh, T17, and NK cells in HS (Figure 3, J and K). Similar to previously published data, we found no difference in the proportion of CD8Tem cells in HS lesional skin compared with healthy skin (16, 17). Marker genes for the identified T cell subtypes are shown in Figure 3L. To identify the nature of the IL-17^+^ T cells, we generated correlation plots between *IL17A*, *CD4*, and *CD8A*, revealing that CD4^+^ T cells and CD8^+^ T cells are likely both a source of *IL17A* in HS lesional skin (Figure 3, M and N). Moreover, these cells were found to express both *IL17A* and *IL17F* (Figure 3O).

As B cells are a prominent component of chronic HS lesions and the formation of tertiary lymphoid structures has been described, the presence of Tfh cells in our HS samples was intriguing (8, 9). Tfh cells are known for their interaction with B cells within lymphoid organs rather than in inflamed peripheral tissue, where this role is normally executed by T peripheral helper (Tph) cells (18). Thus, we assessed the expression of several shared and unique markers of Tfh and Tph lineages to uncover clues to the role of these 2 cell subtypes in HS lesions (Supplemental Figure 3). The clear absence of *CCR2* and *CCR5* expression in this cell cluster supported the annotation of these cells as Tfh rather than Tph cells (Supplemental Figure 3). The expression of *CXCR5* and the Tfh-defining transcription factor *BCL6* in at least a subset of these cells suggests the presence of mature Tfh cells in HS lesional skin (Supplemental Figure 3). These data substantiate a role for this *CXCL13*-expressing T cell population in the previously identified formation of tertiary lymphoid structures in chronic HS lesional skin (Supplemental Figure 3) (8).

### Distinct epidermal KC maturation states in HS reflect different cytokine responses.

**Figure 4:**
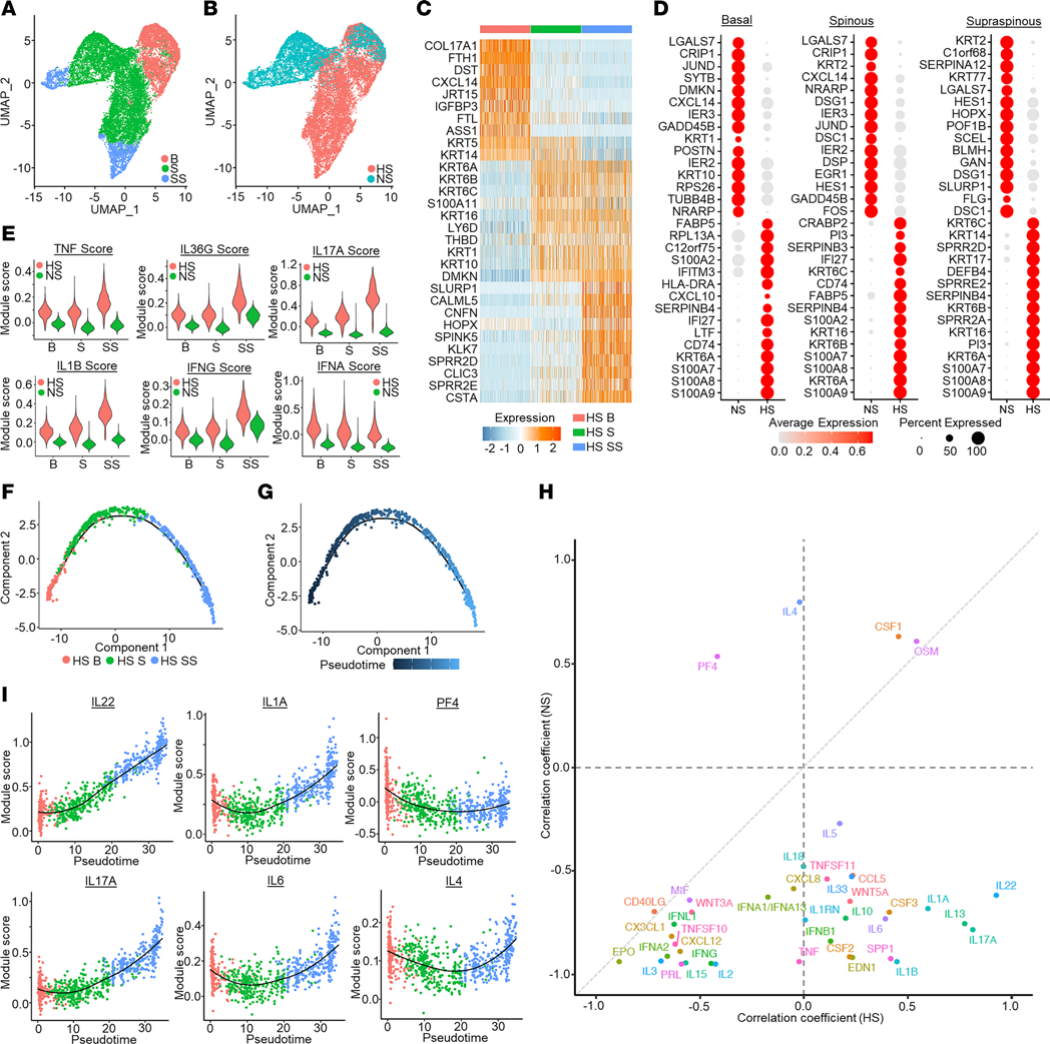
Activation and differentiation pathways of HS KCs are driven by local cytokines. (**A**) UMAP plot showing 16,986 KCs colored by maturation state. B, basal KCs; S, spinous KCs; SS, supraspinous KCs. (**B**) UMAP plot showing the KCs colored by disease condition. (**C**) Heatmap showing marker genes with the highest fold change for each subtype. NS, healthy control. (**D**) Dot plot showing the top 15 differentially expressed genes comparing HS with NS in the basal (left), spinous (middle), and supraspinous (right) layers. The color scale represents the scaled expression, and the size of the dot represents the percentage of KCs expressing each gene. (**E**) Violin plots showing the cytokine module scores in the KC subtypes, split for HS (red) and NS (green). (**F**) Pseudotime trajectory colored by the subtype identity of HS KCs. (**G**) Pseudotime trajectory colored by the pseudotime of the HS KCs. Dark blue represents early; light blue represents late. (**H**) Scatterplot showing the correlation between upstream regulators for HS and NS KCs. (**I**) Scatterplot showing the correlation between HS-derived KC pseudotimes and module scores for IL-17A, IL-22, IL-1A, and IL-6, calculated using genes induced in cultured KCs stimulated by individual cytokines. The color represents the pseudotime subtype identity of the cell.

The clear separation of HS and NS epidermal KCs in Figure 1, A and B, indicates transcriptomic changes in HS KCs suggestive of altered cell function. To further characterize these differences, we subclustered the KC population and annotated basal, spinous, and supraspinous KCs based on marker gene expression (Figure 4, A–C). Next, we performed differential gene expression analysis between HS and NS KCs within each maturation subtype to identify the top distinguishing transcripts (Figure 4D). Across all epidermal layers, HS KCs showed markedly increased expression of antimicrobial/antifungal S100 genes (*S100A7*, *S100A8*, and *S100A9*) and proliferation genes (*KRT6A* and *KRT16*). HS spinous and supraspinous KCs showed a loss of expression of desmosomal cadherins *DSG1* and *DSC1*, as well as *KRT2* (Figure 4D).

We next sought to identify the inflammatory drivers of these subtypes by interrogating cells of each maturation subtype for genes known to be induced in cultured KCs by certain cytokines such as TNF, IL-17A, IL-36γ, and type I IFN (IFN-α). HS KCs showed heightened scores for TNF, IL-17A, IL-36γ, IFN-γ, and type I IFN responses in all 3 maturation subtypes compared with NS skin (Figure 4E). HS KCs showed a striking increase in TNF, IL-1β, and IL-17A response scores from spinous and supraspinous KCs, whereas NS KCs showed a minimal increase. A prominent increase from spinous to supraspinous KCs was also observed for IL-36γ and IFN-γ responses in both HS and NS KCs, albeit on average higher for HS KCs.

To address the distinct separation of the NS and HS KCs, particularly the spinous and supraspinous KCs, we performed pseudotime analyses using Monocle (19) to examine the NS and HS KC maturation pathways separately. This arranged both HS KCs (Figure 4, F and G) and NS KCs (Supplemental Figure 4, A and B) into linear trajectories in the expected direction of basal-spinous-supraspinous maturation. Next, to identify potential cytokines that drive maturation of HS and NS KCs, variable genes along either the NS or HS pseudotime were divided into 5 expression patterns (clusters, HS KCs in Supplemental Figure 4C and NS KCs in Supplemental Figure 4D). We then inferred the upstream regulators for the genes in each cluster using Ingenuity Pathway Analysis (IPA). For each upstream regulator, we calculated a module score using all target genes across the 5 expression patterns/clusters and the correlation between the module scores for each upstream regulator and the pseudotime defined by the Monocle analysis (Figure 4H). These analyses showed that module scores for IL-17A, IL-22, IL-1α, IL-1β, and IL-6 were positively correlated with HS KC pseudotime, whereas IL-4 and PF4 correlated with NS KC pseudotime (Figure 4I). Subsequently, to validate the cytokines driving HS KC maturation, we calculated module scores using genes induced in cultured KCs stimulated by individual cytokines: IL-17A, IL-22, IL-1α, IL-1β, and IL-6. The module scores for these 5 cytokines were highly correlated with the HS but not the NS KC pseudotime, consistent with the results obtained in the IPA analysis (Figure 4I and Supplemental Figure 4E).

Taken together, these results suggest that the altered KC maturation seen in chronic HS lesions is driven by local cytokines, particularly IL-17A, IL-22, IL-1α, IL-1β, and IL-6. Their activation and subsequent functional responses are mainly driven by TNF, IL-17A, IL-36γ, IFN-γ, and type I IFNs.

### Proliferative blood vessels can promote immune cell infiltration in HS chronic lesional skin.

Chronic HS is characterized by a massive influx of immune cells as well as clinically prominent angiogenesis. As expected, IHC and immunofluorescence staining for CD31 (endothelial cells) and ACTA2 (vascular mural cells) showed prominent vascularization of HS chronic skin lesions (Supplemental Figure 5, A and B). Subclustering the endothelial cells identified 5 vascular endothelial clusters (EC0, 1, 2, 4, and 5) and 1 lymphatic endothelial cluster (EC3) (Supplemental Figure 5C). Both EC4 and EC5 were nearly completely derived from HS lesional skin (Supplemental Figure 5, D and E). These HS-associated subclusters showed an immunologically active phenotype, with the expression of immune-activated genes, e.g., *ICAM1*, *SELE*, *IL6*, and *CCL14*. The HS-associated subclusters showed expression of *HLA-DRB5* and *HLA-DRA*, which could allow them to orient the HS T cell response toward a Th17 proinflammatory response (Supplemental Figure 5, F and G) (20). Moreover, EC5 subcluster markers *COL4A1*, *COL4A2*, and *SPARC* are associated with vascular remodeling and angiogenesis (Supplemental Figure 5F). Interrogating the enriched biological processes of the EC4 and EC5 subclusters demonstrated EC4 to be particularly immunologically active (Supplemental Figure 5H). The EC5 subcluster is highly transcriptionally active, showing upregulation of several protein translation processes (Supplemental Figure 5I).

As smooth muscle cells integrate with endothelial cells to form the vasculature, we next examined this cell subset. We identified 6 smooth muscle subclusters with 2 subclusters, SMC0 and SMC6, almost exclusively derived from HS lesional skin (Supplemental Figure 6, A–C). These 2 subclusters both showed expression of *IGFBP4*, *IGFBP2*, *COL4A1*, and *TIMP1*, genes associated with vascular smooth muscle cell proliferation and migration (Supplemental Figure 6D). In addition, SMC6 showed a proinflammatory phenotype with increased expression of *CCL2*, *CXCL2*, and *CXCL3* (Supplemental Figure 6D). Analysis of the enriched biological processes showed both SMC subclusters to be highly transcriptionally active, with SMC6 demonstrating prominent activation via local cytokine stimuli (Supplemental Figure 6, E and F).

In summary, these results support the clinical signs of active vascular proliferation seen in chronic HS lesions and demonstrate the role of immunologically active endothelial cells in the massive infiltration of immune cells in chronic HS lesions.

### Functionally diverse FB subtypes likely drive HS inflammation and fibrosis.

**Figure 5:**
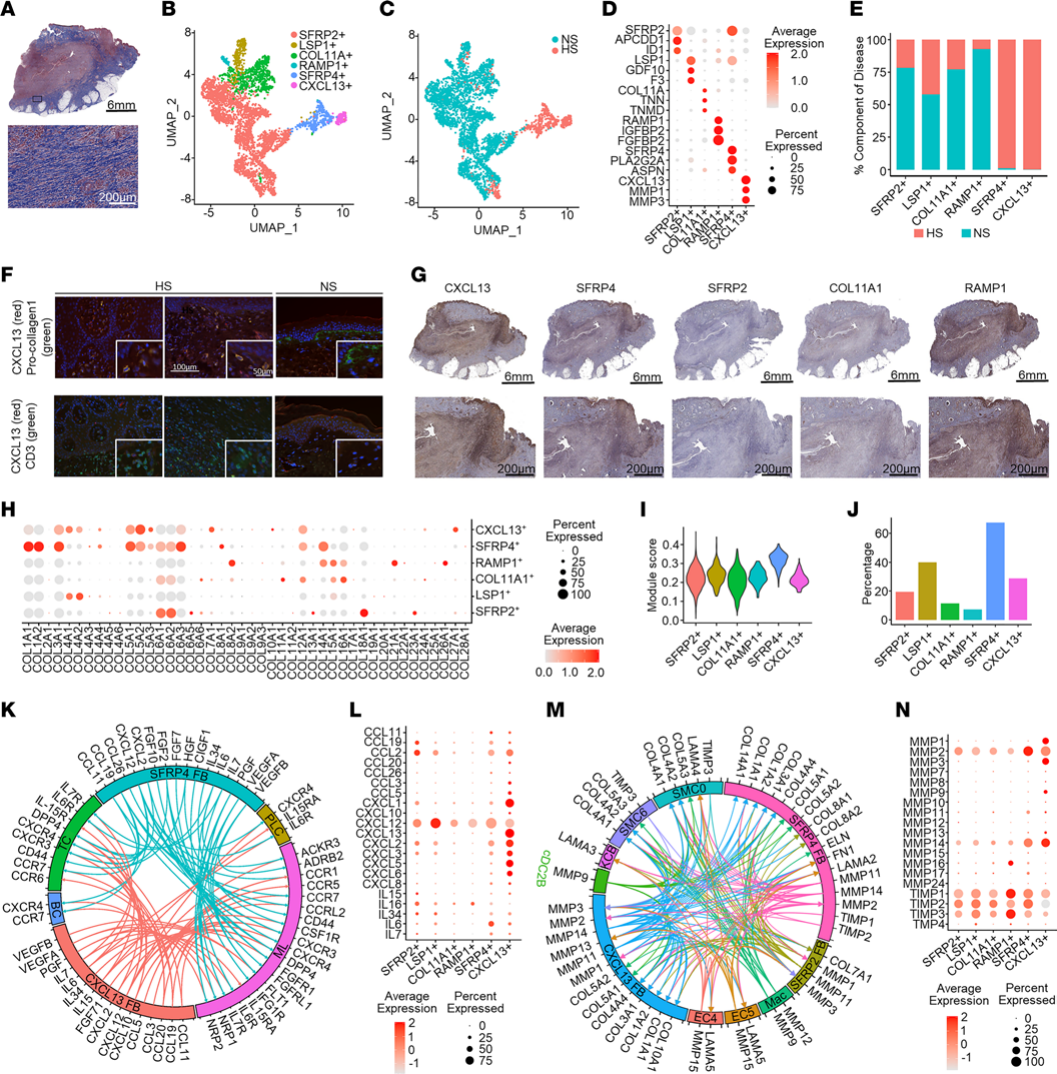
Identification of HS-associated FB subsets. (**A**) Trichrome staining of HS lesional skin (patient HS1). Blue, collagen. Scale bars: top, 6 mm; bottom, 200 μm. (**B**) UMAP plot showing 4,459 FBs colored by subtype: SFRP2^+^, LSP1^+^, COL11A^+^, RAMP1^+^, SFRP4^+^, and CXCL13^+^. (**C**) UMAP plot showing the cells colored by disease condition. NS, healthy control. (**D**) Dot plot showing the representative marker genes for each subtype. Color scale represents scaled expression; size of the dot represents the percentage of cells expressing the gene. (**E**) Bar chart showing the cell types as percentage component of disease. (**F**) Immunofluorescence showing the colocalization of CXCL13 with vimentin (FBs) and to a lesser extent CD3 (T cells). Scale bars: 100 μm; insets, 50 μm. (**G**) IHC showing FB subsets in HS lesional skin (patient HS1). Scale bars: top, 6 mm; bottom, 200 μm. (**H**) Dot plot showing the expression of collagen genes for each FB subtype. Color scale represents scaled expression; size of the dot represents the percentage of cells expressing the gene. (**I**) Extracellular matrix (ECM) module score plotted using ECM pathway gene list from Gene Ontology. (**J**) Expression of ACTA2 among FB subtypes. (**K**) Circos plot showing the cytokine and chemokine interactions from the SFRP4^+^ and CXCL13^+^ FBs with other cell types: PLC, plasma cells; ML, myeloid cells; BC, B cells; TC, T cells. (**L**) Dot plot showing the expression of cytokines and chemokines among the FB subsets. (**M**) Circos plot representing the interactions of MMPs, collagens, and laminins between the most prominent HS-associated cell subtypes: Mac, macrophages; EC4, endothelial cell subcluster 4; EC5, endothelial cell subcluster 5; cDC2B, classical type 2 dendritic cell subset B; SMC6, smooth muscle cell subcluster 6. (**N**) Dot plot showing the expression of MMPs among the FB subsets.

While extensive fibrosis is a hallmark of chronic HS, as demonstrated by trichrome staining (Figure 5A), FBs have not been studied in detail (2, 12, 13). Therefore, we aimed to further characterize the differences between HS and NS FBs. We identified 11 clusters, which we annotated into 6 FB subtypes according to previously published marker genes: *SFRP2^+^* (secreted frizzled-related protein 2), *LSP1^+^* (lymphocyte-specific protein 1), *COL11A^+^* (collagen type XI α1 chain), *RAMP1^+^* (receptor activity–modifying protein 1), *SFRP4^+^*, and *CXCL13*^+^ (C-X-C motif chemokine ligand 13) FBs (Figure 5B) (21). Two of these subtypes, *SFRP4^+^* and *CXCL13^+^* FBs, were derived nearly exclusively from HS samples (Figure 5C). The top 3 marker genes for all FB subtypes are shown in Figure 5D. The *SFRP4^+^* and *CXCL13*^+^ FBs were not only specifically derived from HS samples but were also found in a profoundly increased proportion compared with the other FB subtypes in these samples (Figure 5E). Quantitative PCR corroborated increased expression of specific marker genes of these populations in primary FBs derived from lesional HS versus NS skin (Supplemental Figure 7A). Costaining of CXCL13 and either vimentin (FB marker) or CD3 (T cell) by immunofluorescence demonstrated more prominent protein expression of CXCL13 among FBs than T cells in HS lesional skin (Figure 5F). IHC further confirmed the presence of the identified FB subtypes in HS lesional skin (Figure 5G). Both the *CXCL13*^+^ and the *SFRP4*^+^ FBs were found to demarcate the edges of the inflammatory infiltrate (Figure 5G and Figure 1F). Dot, violin, and feature plots of expression levels of the most prominently expressed collagen genes revealed the strongest expression among the *SFRP4^+^* FBs (Figure 5H and Supplemental Figure 8, A and B). Taken together with a high extracellular matrix (ECM) module score (Figure 5I) and high expression of *ACTA2* (actin α2, smooth muscle; Figure 5J), *SFRP4^+^* FBs were identified as myofibroblasts.

To further characterize the functions of these HS-associated *CXCL13*^+^ and *SFRP4*^+^ FBs, we performed analysis of upregulated canonical pathways and enriched Gene Ontology biological processes. As expected, the *SFRP4^+^* subtype showed functions associated with fibrosis and ECM formation (Supplemental Figure 8, C and D). Additionally, this subtype demonstrated immunological functions enriched for neutrophil activation. Canonical pathway analysis of the *CXCL13^+^* FBs identified numerous upregulated signaling pathways, most prominently pathways associated with oncostatin M (OSM) and IL-17A/F (Supplemental Figure 8E). Upregulated biological processes showed these cells to be highly transcriptionally active, with immunological functions aimed at attracting and activating neutrophils and lymphocytes (Supplemental Figure 8F). Ligand-receptor analysis for chemokines and cytokines expressed by the *SFRP4*^+^ and *CXCL13*^+^ FBs revealed that both subtypes were engaged in extensive communication networks with different immune cells within the HS infiltrate (Figure 5K), although the expression of these cytokines and chemokines was highest in the *CXCL13^+^* FBs (Figure 5L). Furthermore, the *SFRP4*^+^ and *CXCL13*^+^ FBs contributed to a complex interplay among different MMPs, collagens, and laminins derived from the distinct HS-associated cell subtypes to promote ECM deposition and remodeling (Figure 5, M and N). Taken together, these data support a prominent proinflammatory and remodeling role for the *CXCL13^+^* FBs and implicate *SFRP4^+^* FBs as myofibroblasts, with a prominent expression of *COL1A1* and *ACTA2*, driving fibrosis in chronic HS.

**Figure 6:**
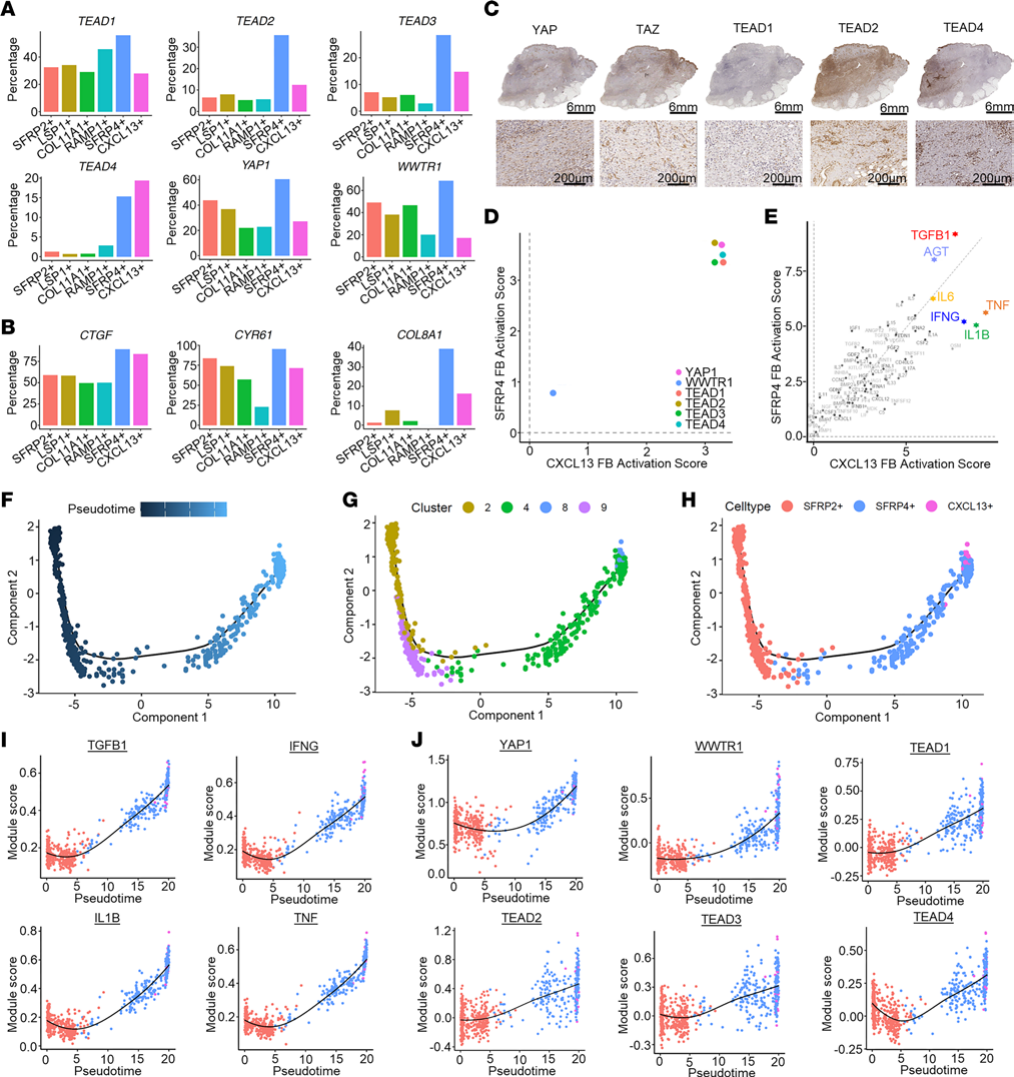
Expression of Hippo pathway genes and their association with HS FB pseudotime. (**A** and **B**) Percentage of FB subtypes expressing Hippo pathway marker (**A**) and target genes (**B**). (**C**) IHC showing localization of Hippo pathway marker genes (patient HS1). Scale bars: top, 6 mm; bottom, 200 μm. (**D** and **E**) Scatterplots showing the activation *z* scores of Hippo pathway marker genes (**D**) and activated cytokine and growth factor upstream regulators (**E**) as upstream regulators for the *SFRP4^+^* and *CXCL13^+^* FBs. (**F**) Pseudotime trajectory of HS *SFRP2^+^*, *SFRP4^+^*, and *CXCL13^+^* FBs colored by the pseudotime. Dark blue represents early, light blue represents late pseudotime. (**G**) Pseudotime trajectory colored by the pseudotime subcluster of the FBs. (**H**) Pseudotime trajectory colored by the subtype identity of HS FBs. (**I** and **J**) Scatterplots showing the correlation between the FB pseudotimes and module scores for previously identified upstream regulators (**I**) and Hippo pathway–associated genes (**J**). The color represents the pseudotime subcluster identity of the cell.

Recent clinical and preclinical studies have implicated the contribution of Hippo signaling pathway components in fibrotic diseases in many organs, including the lung, heart, and skin (22–24). To investigate the role of Hippo pathway signaling in HS fibrosis, we assessed the expression of Hippo pathway signaling factors in our FB subsets. This revealed increased expression of both Hippo pathway transcriptional coactivators and transcription factors (*YAP1*, *WWTR1*, and *TEAD1–TEAD4*) (Figure 6A) as well as known target genes (*CTGF*, *CYR61*, and *COL8A1*) (Figure 6B) primarily among the *SFRP4^+^* population. Protein expression of YAP, WWTR1/TAZ, TEAD1, TEAD2, and TEAD4 was confirmed in HS lesional skin FBs by IHC (Figure 6C).

To further support the hypothesis that Hippo pathway signaling is involved in the activation of HS myofibroblasts, we performed upstream regulator analysis. Indeed, in addition to well-known profibrotic markers such as TGF-β and angiotensinogen (AGT, which has previously been identified as a critical component in cardiac and pulmonary fibrosis; refs. 21, 25), we identified several factors belonging to the Hippo pathway (*YAP1*, *WWTR1*, and *TEAD2*), particularly among the *SFRP4^+^* myofibroblasts (Figure 6D and Supplemental Figure 9A). In addition, the key HS-associated cytokines TNF, IL-1β, IFN-γ, and IL-6 were found to be highly activated upstream regulators for both the *CXCL13^+^* and *SFRP4^+^* subtypes (Supplemental Figure 9B).

Next, we performed pseudotime analysis to identify whether Hippo pathway transcription factors were associated with the activation and development of the *SFRP4^+^* and *CXCL13^+^* FB phenotypes, using the underlying identified clusters (Figure 6E). These clusters were arranged into a linear trajectory in the direction from the *SFRP2^+^* to *SFRP4^+^*, with a less clearly defined *CXCL13^+^* endpoint (Figure 6, F–H). Not only *TGFB*, *TNF*, *IFNG*, and *IL1B* (Figure 6I) but also the Hippo pathway transcriptional regulator *YAP*, its coactivator *WWTR1*, and transcription factors *TEAD1–4* (Figure 6J) were found to be highly correlated with the FB pseudotime. Interrogating ATAC-Seq (assay for transposase-accessible chromatin using sequencing) data demonstrated increased chromatin accessibility in the *WWTR1*, *TEAD1*, and *COL8A1* regions of lesional HS FBs compared with nonlesional and healthy skin FBs (Supplemental Figure 10), further supporting the activation of the Hippo pathway in HS lesional FBs.

**Figure 7:**
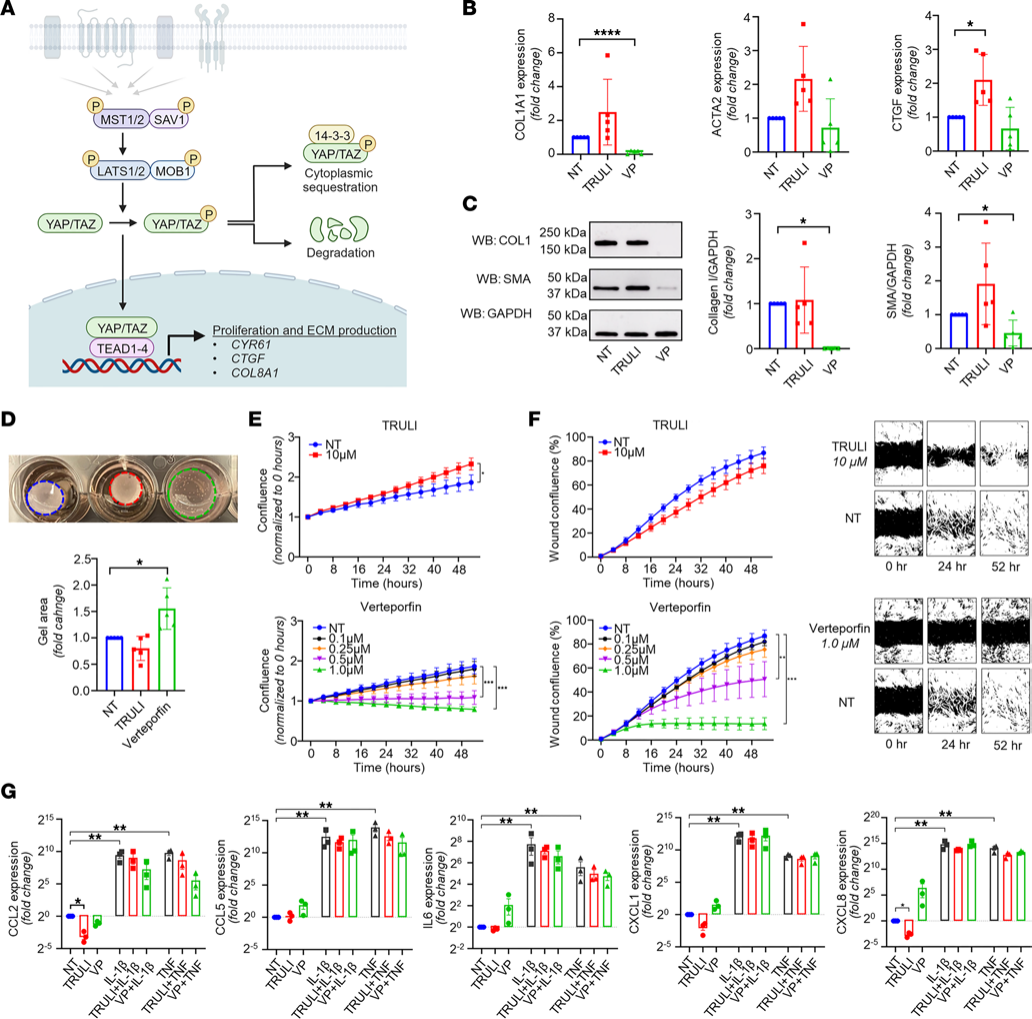
Modulation of the Hippo pathway in primary HS FBs. (**A**) Illustration of Hippo pathway, created with BioRender (biorender.com). (**B**) Quantitative PCR results showing the effect of TRULI or verteporfin (both 10 μM) on ACTA2, COL1A1, and CTGF expression in HS FBs (*n* = 5; *P < 0.05, ****P < 0.0001; mean ± SD; ANOVA/Kruskal-Wallis test). (**C**) Effect of TRULI or verteporfin (both 10 μM) on smooth muscle actin (SMA) and collagen I levels in HS FBs by Western blotting (*n* = 5; *P < 0.05; mean ± SD; Kruskal-Wallis test [collagen I], ANOVA [SMA]). (**D**) Verteporfin blocked gel contraction in HS FBs. Data normalized to the corresponding NT (untreated) group (*n* = 5; *P < 0.05; mean ± SD). (**E**) TRULI significantly increased cell proliferation while verteporfin dose-dependently blocked cell growth among HS FBs (*n* = 3; *P < 0.05, ***P < 0.0001; mean ± SEM; 2-way repeated-measures ANOVA). The same NT group is shown in both panels. Cell proliferation was monitored by analysis of the area occupied by cells over time, using IncuCyte S3 Analysis software. (**F**) Verteporfin showed a dose-dependent reduction in cell migration of HS FBs (*n* = 3; **P < 0.01, ***P < 0.001; mean ± SEM; 2-way repeated-measures ANOVA). The same NT group is shown in both panels. (**G**) Expression of cytokines and chemokines among untreated (NT), IL-1β–stimulated (10 ng/mL), and TNF-α–stimulated (10 ng/mL) primary HS FBs treated or not treated with TRULI or verteporfin (*n* = 5; *P < 0.05, **P < 0.01, ***P < 0.001; mean ± SD; 1-way repeated-measures ANOVA).

To uncover the functional role of Hippo signaling (Figure 7A) in HS fibrosis, we performed ex vivo experiments using primary dermal FBs obtained from chronic HS lesions. FBs were stimulated with either TRULI (which blocks YAP phosphorylation, thereby activating YAP-mediated transcriptional coactivation; ref. 26) or verteporfin (which disrupts YAP-TEAD interaction, resulting in YAP target inhibition; ref. 27). Verteporfin significantly reduced both protein and RNA expression of collagen I and, to a lesser extent, smooth muscle actin (SMA/*ACTA2*) in HS FBs (Figure 7, B and C). Verteporfin stimulation also significantly inhibited HS FB contractility in the gel contraction assays (Figure 7D) and resulted in a significant dose-dependent reduction of both proliferation and migration of HS FBs (Figure 7, E and F). In contrast, stimulation of YAP transcriptional activity with TRULI resulted in a nonsignificant increase in RNA expression of smooth muscle actin (*ACTA2*) and collagen I (*COL1A1*) (Figure 7B). TRULI treatment did significantly induce *CTGF* expression (Figure 7B). Treatment with TRULI also significantly increased proliferation but failed to further increase either migration or gel contraction (Figure 7, D–F). Performing the same experiments with healthy control FBs showed similar results upon TRULI or verteporfin stimulation in comparison with HS FBs, but to a lesser extent (Supplemental Figure 11). In particular, upregulation of this pathway by TRULI seemed to result in a more limited upregulation of collagen I or smooth muscle actin RNA and protein in comparison with HS FBs (Figure 7B and Supplemental Figure 11, A and B). Moreover, TRULI was unable to induce further proliferation of healthy FBs, which was already significantly lower than that of HS FBs.

To assess the relevance of the Hippo pathway to proinflammatory characteristics of HS FBs, we examined the expression of several cytokines and chemokines after TRULI and verteporfin stimulation alone or in combination with single cytokine stimulations. Overall, neither TRULI nor verteporfin significantly affected the expression of *CCL2*, *CCL5*, *CXCL1*, *CXCL8*, or *IL6* in HS FBs in response to stimulation with the previously identified upstream regulators IL-1β, TNF, or IFN-γ (Figure 7G). These experiments indicate that the Hippo pathway is involved in HS myofibroblast differentiation but dispensable for the HS-specific *CXCL13^+^* FB phenotype.

Taken together, these data support a role for the Hippo pathway in promoting the extensive fibrosis of HS and demonstrate that inhibition of this pathway can modulate the profibrotic characteristics of HS FBs, independent of their proinflammatory characteristics.

### Ligand-receptor analysis reveals cell subtype–specific networks in HS lesional skin.

**Figure 8:**
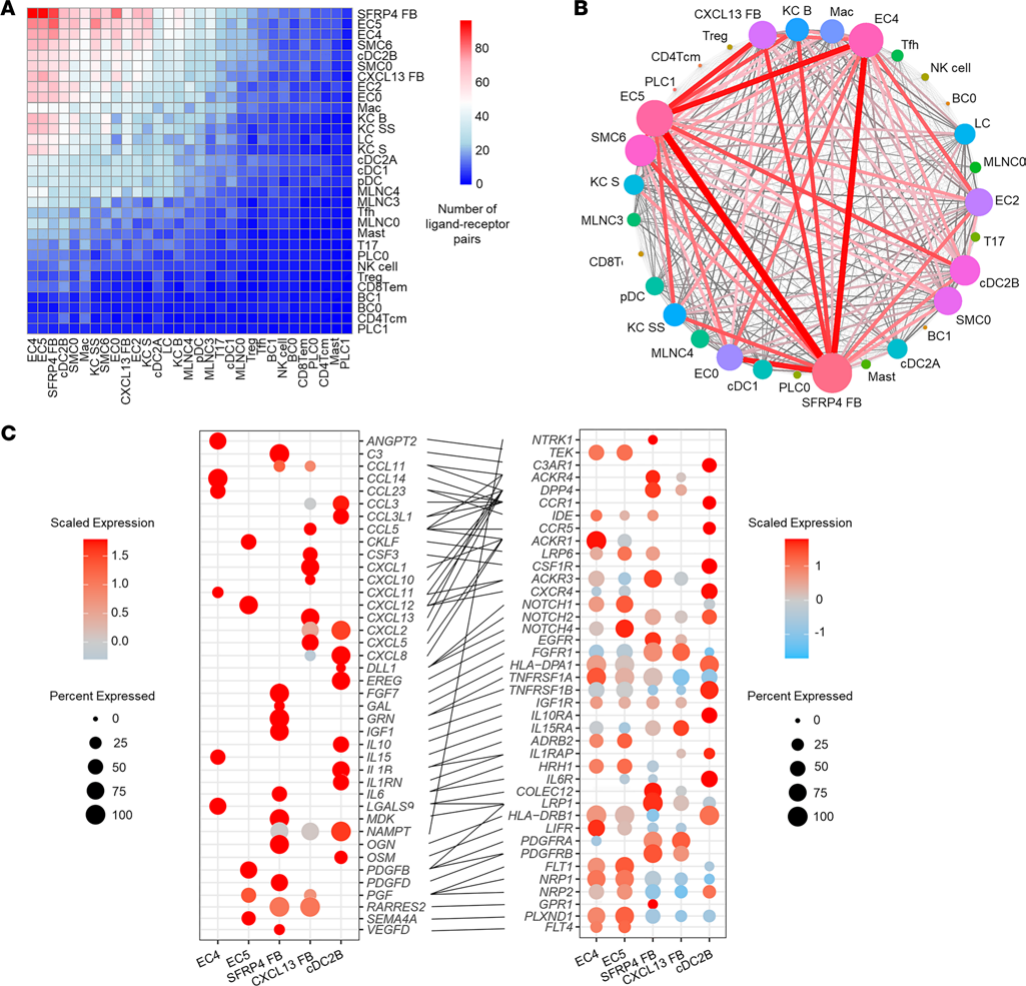
Ligand-receptor analysis reveals cell subtype–specific networks in HS lesional skin. (**A**) Heatmap showing the number of ligand-receptor pairs with a higher score in HS compared with NS among the previously identified cell subtypes. The ligands were expressed by the cell types in the row, and the receptors were expressed by the cell types in the column. The color scale represents the number of ligand-receptor pairs. (**B**) Connectome web showing ligand-receptor interactions between all identified cell subsets. Thickness of a line indicates the number of interactions. (**C**) Dot plot showing selected ligand-receptor interactions between the 5 most contributing cell subtypes. The color scale represents the scaled expression of the gene. The size of the dot represents the percentage of cells expressing the gene of interest; lines link the ligands to receptors.

Given the marked shifts in cell subtype composition in chronic HS lesional skin, we analyzed the cell-cell communication between cell subtypes in HS skin. Intriguingly, the greatest number of ligand-receptor pairs were found for the *SFRP4*^+^ FB subtype, particularly in connection with the EC4 and EC5 endothelial cell subsets (Figure 8, A and B). Plotting the expression of their ligands and receptors demonstrates how *SFRP4*^+^ FBs express *VEGFD*, *FGF7*, and *IGF1*, providing strong angiogenic stimuli to both the immunologically active EC4 and transcriptionally active EC5 subtypes (Figure 8C). In line with its proinflammatory phenotype, the *CXCL13*^+^ FB subtype was found to express a multitude of angiogenic chemokines: *CCL3*, *CCL5*, *CXCL1*, *CXCL5*, and *CXCL8* (28, 29). In turn, EC4 and EC5 use distinct signaling molecules to communicate with the FB subtypes. EC5 expresses *SEMA4A* and *PDGFB*, promoting proliferation and profibrotic characteristics in FBs (30, 31). In contrast, the EC4 subcluster expresses *CXCL11* and *IL15*, which have been demonstrated to have antifibrotic properties in several animal models of fibrotic disease (28, 32). Additionally, the EC subclusters also express either *CCL14* or *CXCL12*, which bind to their respective receptors *CCR1* and *CXCR4* on cDC2B cells, facilitating their transendothelial migration and aiding survival (33). These cDC2B cells in turn communicate with both endothelial cell clusters through *CXCL8*, *IL1B*, and *CCL3* to promote angiogenesis and increase vascular permeability (34). Interestingly, cDC2B cells also express *DLL1*, which binds to *NOTCH* receptors present on all endothelial and FB subtypes to promote angiogenesis and collagen release, respectively (35).

In summary, HS lesional skin hosts complex cellular crosstalk in which cDC2B cells stimulate endothelial cells and FBs, which in turn attract and activate cDC2B cells, ultimately resulting in a dense immune infiltrate accompanied by extensive fibrosis and angiogenesis.

## Discussion

Here, through a combination of scRNA-Seq, spatial transcriptomics, and immunostaining, we provided several critical insights into the pathogenesis of HS. We reveal a highly structured and compartmentalized inflammatory response in chronic HS, and we demonstrate how this compartmentalization is orchestrated through cellular crosstalk between immune cells and stromal cells. We further establish that 2 stromal subtypes enriched in HS lesional skin, *CXCL13*^+^ and *SFRP4*^+^ FBs, play a major role in shaping and perpetuating the inflammatory response in HS through secretion of chemokines that recruit B cells and myeloid cells, as well as driving the extensive fibrosis that is characteristic of long-standing HS.

Our data characterize the cellular crosstalk likely responsible for immune compartmentalization in HS skin. At the center of HS lesions, including abscesses and sinus tracts, neutrophils are found in close proximity around ruptured tunnel fragments (Figure 1, F and H) (2). Here, they likely represent the first line of defense in response to damage-associated molecular patterns, pathogen-associated molecular patterns, and complement factors (2, 36). Their primary antimicrobial functions of phagocytosis, degranulation, and the release of neutrophil extracellular traps (NETs) result in the characteristic purulent drainage from abscesses and tunnels (37). We found other immune cell populations such as cDC2B cells, pDCs, macrophages, and T cells near the neutrophil infiltration (Figure 1, F and H, and Figure 3E). Both cDC1 and cDC2 subtypes contribute to the respective induction of Th1 and Th17 subtypes (38), the latter of which was enriched in HS in our data (Figure 3K), consistent with previous observations. In contrast, B cells were found primarily at the edges of the infiltrate near the demarcating layer primarily consisting of *CXCL13*^+^ and *SFRP4^+^* FBs (Figure 1, F and H, and Figure 5G). In addition, *CXCL13*^+^ FBs expressed multiple other chemokines (i.e., *CXCL12*, *CCL19*), which likely further contribute to the spatial localization of the B cell population at the periphery of actively inflamed abscesses and sinus tracts. This crosstalk is likely bidirectional, with our previous study demonstrating the expression of, e.g., *TGFB1* by B and plasma cells in chronic lesional skin (9). Moreover, B cells have also been shown to be able to directly induce fibrosis in patients with IgG4-related disease (39). *SFRP4^+^* myofibroblasts were found at the edge of the lesions, close to B cell populations, contributing to the fibrotic zone surrounding the actively inflamed areas in the skin. Within this zone, clusters of B cells, T cells, and plasma cells were found (Figure 1, F and H), suggestive of tertiary lymphoid-like structures (TLSs).

These TLSs have previously been described in chronic HS lesions, and our current data suggest that their formation might be in part driven by *CXCL13^+^* FBs (8). TLS formation involves recruitment and homing of T cells through CCL19 and CCL21, and chemoattraction and maintenance of B cells through CXCL13-CXCR5 interactions (40, 41). Our data support such a mechanism in HS, with both *CCL19* and *CXCL13* being expressed by the HS-enriched *CXCL13^+^* FB subtype (Supplemental Figure 8, E and F). Differentiation of TLS-associated FBs is a known phenomenon in response to inflammatory triggers such as TNF, IL-17, and IL-23 (42, 43). These cytokines play a prominent role in the pathogenesis of HS (2), and both TNF and, to a lesser extent, IL-17 were identified as upstream regulators of *CXCL13^+^* activation in our data. Remarkably, a large proportion of *CXCL13^+^* FBs showed higher expression of *CXCL13* than Tfh cells, likely reflecting the importance of the *CXCL13^+^* FB subtype to the migration of B cells, and their spatial localization at the periphery of the actively inflamed areas of chronic HS lesions, potentially as TLSs. In addition, the expression of *CXCL12* and *IL7* by *CXCL13^+^* FBs (Supplemental Figure 8, E and F) may contribute to chemotaxis and survival of both B and T cells in HS lesions. TLSs actively regulate local immune responses, influence disease progression, and likely contribute to the large number of B and plasma cells present in chronic HS lesions (9, 44), potentially making them a therapeutic target in HS. Furthermore, *CXCL13^+^* FBs may further promote inflammatory responses through the expression of a wide range of cytokines and chemokines, including the neutrophil chemokines *CXCL1*, *CXCL2*, and *CXCL8* (Supplemental Figure 8, E and F), which in turn may promote NETosis, a prominent feature of HS inflammation (37, 45). In addition, this population demonstrated the most prominent expression of multiple MMPs, likely contributing to tissue destruction through proteolysis of epithelial cell junction proteins and regulation of cell-matrix interactions. Moreover, MMPs may play a role in the immune response in HS through regulating cytokine and chemokine activity and gradient formation (46). This broad inflammatory contribution of *CXCL13^+^* FBs to HS pathogenesis identifies this subtype as a potential target to alter the chronic inflammatory response in HS.

In addition to prominent immune cell infiltration, fibrosis is a hallmark of long-standing HS. Our study implicates another HS-associated FB subtype in this process: the *SFRP4*^+^ myofibroblasts, whose primary function, the production of extracellular matrix (ECM) components, was found to be driven by Hippo pathway signaling, a profibrotic pathway in HS pathogenesis. The Hippo pathway is a highly conserved pathway that has been shown to play a central role in regulating cell proliferation and tissue regeneration (47). Increased activation of this pathway has been shown to play a pivotal role in fibrotic diseases such as idiopathic pulmonary fibrosis (22), and our data further implicate this pathway in HS fibrosis (Figure 7). Central to Hippo signaling is a kinase cascade, wherein MST1/2 and SAV1 form a complex to phosphorylate and activate LATS1/2 (Figure 7A) (48). LATS1/2 kinases in turn phosphorylate the transcriptional coactivators YAP and TAZ, resulting in sequestration of the YAP/TAZ complex in the cytoplasm and subsequent degradation. When dephosphorylated, however, the YAP/TAZ complex translocates into the nucleus, where it interacts with the transcription factors TEAD1–4 to promote the expression of multiple genes associated with cell proliferation, myofibroblast development, and collagen deposition (48). In HS FBs, treatment with verteporfin, which inhibits transcriptional activity of the Hippo pathway through disruption of the interaction between YAP/TAZ and TEAD1–4, reduced both the myofibroblast phenotype and collagen production, whereas the opposite response was seen with TRULI, which promotes translocation of YAP into the nucleus, promoting binding with TEAD transcription factors (Figure 7, B–F). Notably, however, Hippo pathway modulation had minimal effect on proinflammatory responses of HS FBs, suggesting that fibrosis can be uncoupled from the inflammatory response in HS (Figure 7G).

Currently, compounds are in development targeting the Hippo pathway for both the treatment of cancer (though inhibition of YAP/TAZ) and wound healing and tissue regeneration (through activation of YAP/TAZ) (23). A recent mouse study showed how activation of Gα-coupled dopamine receptor D1 inhibits YAP/TAZ function in mesenchymal cells, reversing in vitro ECM stiffening and in vivo lung and liver fibrosis (49). This demonstrates that these compounds can potentially be leveraged for use in fibrotic diseases, potentially providing future treatment options for extensive and debilitating HS-associated fibrosis. Ultimately, treatment of HS is likely to be a combination of compounds with antiinflammatory and potentially antifibrotic effects.

Our study does have several limitations. First, owing to the scRNA-Seq protocol used, we were unable to efficiently capture neutrophils in our scRNA-Seq analysis. Neutrophils are known to play an important role in the pathogenesis of HS, which was supported by the expression of a wide range of neutrophil-attracting and activating molecules by several different cell types. In line with this, to generate single-cell data, tissue is removed from its microenvironment and subjected to several lysis steps and mechanical stress, potentially altering the gene expression of the cells. This highlights the importance of substantiating scRNA-Seq findings by in situ methods such as spatial transcriptomics and IHC. Finally, the samples used and therefore the results found in this study are representative of only a subset of patients with HS: those with moderate to severe disease characterized by chronic inflammation, tissue destruction, and fibrosis. Future studies including samples from both acute and chronic lesions could help elucidate the pathways involved in disease onset and progression and identify valuable new therapeutic targets across the HS disease timeline.

Taken together, the data presented here provide an unprecedented view of the pathogenesis of chronic HS, characterize the main cellular players, and define their interactions. They describe a striking layering of the chronic HS infiltrate and identify the contribution of FB subtypes in orchestrating this compartmentalized immune response. They further demonstrate the central role of the Hippo pathway in promoting the extensive fibrosis characteristic of HS and provide preclinical evidence that the profibrotic FB response in HS can be modulated through inhibition of this pathway. These data provide insights into key aspects of HS pathogenesis with broad therapeutic implications.

## Methods

### Human skin samples for single-cell analyses.

Five patients with chronic HS and 8 healthy controls were recruited for single-cell analysis at the University of Michigan. HS patients had a disease duration of at least 1 year prior to sampling and Hurley stage II or III disease. Patients did not use biologics or i.v. treatment and were off any other systemic treatment and off any topical agents for at least 2 weeks prior to inclusion. Six-millimeter punch biopsies were taken from lesional skin in the case of HS patients and healthy control skin from the hip/buttock for healthy controls.

### scRNA-Seq library preparation, sequencing, and alignment.

Generation of single-cell suspensions for scRNA-Seq was performed on 6 mm biopsies obtained from HS and healthy donors. Samples were incubated overnight in 0.4% dispase (Life Technologies) in Hanks balanced saline solution (Gibco) at 4°C. Epidermis and dermis were separated. Epidermis was digested in 0.25% trypsin/EDTA (Gibco) with 10 U/mL DNase I (Thermo Fisher Scientific) for 1 hour at 37°C, quenched with FBS (Atlanta Biologicals), and strained through a 70 μm mesh. Dermis was minced, digested in 0.2% collagenase II (Life Technologies) and 0.2% collagenase V (MilliporeSigma) in plain medium for 1.5 hours at 37°C, and subsequently strained through a 70 μm mesh. Epidermal and dermal cells were combined at a 1:1 ratio, and libraries were constructed by the University of Michigan Advanced Genomics Core on the 10X Chromium system with chemistry v2 and v3. Libraries were then sequenced on the Illumina NovaSeq 6000 sequencer to generate 150 bp paired-end reads. Data processing including quality control, read alignment (hg38), and gene quantification was conducted using the 10X Cell Ranger software. The samples were then merged into a single expression matrix using the cellranger aggr pipeline. See Supplemental Methods for information on cell clustering, cell type annotation, ligand-receptor analysis, pseudotime trajectory construction, and spatial transcriptomic analyses.

### Isolation of primary dermal FBs.

HS FBs were cultured from routinely excised chronic lesional skin at the Erasmus University Medical Center, Rotterdam, the Netherlands (Supplemental Table 1). FBs were obtained by dissection and mincing of the dermis from excised skin. Minced tissue was placed in DMEM (Lonza BioWhittaker) containing 20% FBS (Gibco; vol/vol), l-glutamine (mM), and penicillin/streptomycin (Lonza BioWhittaker; 10.000 U) and incubated in 5% CO_2_ at 37°C until FB colony formation was observed. At 75%–80% confluence, the FBs were trypsinized with a trypsin/EDTA solution (catalog CC-5012, Lonza) and incubated at 37°C for 5–10 minutes. Trypsin was blocked with DMEM containing 10% FBS and centrifuged at 380*g* for 10 minutes at room temperature for subsequent subculture or cryopreservation. Cryopreserved HS FBs with passage numbers ≤3 were shipped to the University of Michigan and used for functional experiments. In addition, healthy donors were recruited from the Department of Dermatology of the University of Michigan, and dermal FBs were isolated from punch biopsies from the hip/buttock. Healthy controls were age- and sex-matched to the HS patients. Gene expression changes between healthy donor and HS FBs were analyzed by quantitative PCR after total RNA was extracted using the RNA plus easy mini kit (QIAGEN) and cDNA was synthesized with the Applied Biosystems High-Capacity cDNA Reverse Transcription Kit. Quantitative PCR was performed in a 7900HT Fast Real-Time PCR System.

### FB treatment and functional experiments.

Dermal FBs from HS patients were treated with 10 μM of the LATS kinase inhibitor TRULI/Lats-IN-1 (MedChemExpress HY-138489) or 0.1–10 μM of the YAP/TEAD inhibitor verteporfin (Cayman Chemical 17334) for 48–72 hours. Additional 6-hour cytokine stimulations were performed using IL-1β (10 ng/mL; R&D Systems 201-LB-005) and TNF-α (10 ng/mL; R&D Systems 210-TA-005). Gene expression changes in cells were performed by quantitative PCR after total RNA was extracted using Direct-zol RNA MiniPrep Kit (Zymo Research R2052). Quantitative PCR was performed in a ViiA 7 Real-Time PCR System (Applied Biosystems). Protein expression changes were monitored using Western blotting. After blocking, the blots were probed with antibodies against collagen I (COL1, Abcam ab6308) or α-smooth muscle actin (Abcam ab5694). For loading control, the blots were immunoblotted with antibodies against GAPDH (Cell Signaling 2118). Band quantification was performed using ImageJ (NIH) (50). The IncuCyte Live-Cell Imaging System was used to monitor cell proliferation or migration. After addition of different treatments, cells were monitored by IncuCyte. Cell counts were analyzed by the IncuCyte S3 Analysis software. Gel contraction assays were performed using the cell contraction kit from Cell Biolabs (CBA-201).

### Statistics.

For the in vitro experiments, normality was assessed using the Shapiro-Wilk test. To determine the differences between groups, 1-way ANOVA (post hoc Dunnett’s test) or Kruskal-Wallis tests (post hoc Dunn’s test) were performed. For time curve experiments, a repeated-measures 2-way ANOVA (with post hoc 2-stage step-up method of Benjamini, Krieger, and Yekutieli [ref. 51] to control the false discovery rate) was performed. All analyses were performed using GraphPad Prism version 8 (GraphPad Software Inc.). Tests were 2-sided, and *P* values less than 0.05 were considered statistically significant.

### Study approval.

The study was approved by the University of Michigan institutional review board (HUM00174864), and all patients provided written, informed consent.

### Data availability.

The scRNA-Seq data discussed in this publication were deposited in the NCBI’s Gene Expression Omnibus (GEO) and are accessible through GEO Series accession numbers GSE154775 and GSE173706 (https://www.ncbi.nlm.nih.gov/geo/query/acc.cgi?acc=GSE154775; https://www.ncbi.nlm.nih.gov/geo/query/acc.cgi?acc=GSE173706). Data from other experiments and analyses used to generate the figures can be found in the Supporting Data Values file.

## Author contributions

KRVS and JEG conceptualized the study. KRVS, PST, OP, MGK, XX, MKS, RU, MN, ME, CW, CD, JHK, JCB, WDB, and MNM provided investigation. KRVS, FM, PST, RW, and LCT provided formal analysis. KRVS, FM, RW, and LCT provided visualization. KRVS, LCT, JEG, and JMK acquired funding. JEG provided supervision. KRVS and JEG wrote the original draft of the manuscript KRVS, FM, PST, OP, MGK, MC, XX, MKS, RU, PWH, RW, LN, MN, ME, CW, CD, JHK, JCB, WDB, KW, ÖU, MNM, MP, RLM, EM, RS, JMK, ACB, LCT, and JEG wrote, reviewed, and edited the manuscript.

## Supplementary Material

Supplemental data

Supporting data values

## Version 1 12/05/2023

In-Press Preview

## Version 2 02/01/2024

Electronic publication
