# Supplementary material for: Single-cell sequencing reveals Hippo signaling as a driver of fibrosis in hidradenitis suppurativa
Source: J Clin Invest. 2024 Feb 1;134(3):e169225. doi: 10.1172/JCI169225 (PMC10836805; doi:10.1172/JCI169225)
Supplement: Supplemental data [file jci-134-169225-s042.pdf]

SUPPLEMENTAL METHODS

Cell clustering and cell type annotation

The R package Seurat (v3.1.2) was used to cluster the cells in the merged matrix [1]. Cells with less than 500 transcripts or 100 genes, or more than 10% of mitochondrial expression were first filtered out as low-quality cells. The NormalizeData was used to normalize the expression level for each cell with default parameters. The FindVariableFeatures function was used to select variable genes with default parameters. The FindIntegrationAnchors and IntegrateData functions were used to integrate the samples prepared using different 10X Chromium chemistries. The ScaleData function was used to scale and center the counts in the dataset. Principal component analysis (PCA) was performed on the variable genes, and the first 20 PCs were used for cell clustering and Uniform Manifold Approximation and Projection (UMAP) dimensional reduction. The clusters were obtained using the FindNeighbors and FindClusters functions with the resolution set to 0.5. The cluster marker genes were found using the FindAllMarkers function. The cell types were annotated by overlapping the cluster markers with the canonical cell type signature genes. To calculate the disease composition based on cell type, the number of cells for each cell type from each disease condition were counted. The counts were then divided by the total number of cells for each disease condition and scaled to 100 percent for each cell type. Differential expression analyses between NS and HS or any two groups of cells were carried out using the FindMarkers function.

Cell type subclustering

Subclustering was performed on the abundant cell types. The same functions described above were used to obtain the subclusters. Subclusters that were defined exclusively by mitochondrial gene expression, indicating low quality, were removed from further analysis. The subtypes were annotated by overlapping the marker genes for the subclusters with the canonical subtype signature

genes. Ingenuity pathway analysis was applied to the differentially expressed genes to determine the canonical pathways and the potential upstream regulators. The upstream regulators with an activation z score >=2 were considered significantly activated. The module scores were calculated using the AddModuleScore function on the genes induced by the intended cytokine from bulk RNA-seq analysis. To obtain the cytokine induced genes, healthy donor derived keratinocytes and fibroblasts were cultured and stimulated with respective cytokine for 8 hours [2]. Bulk RNA-seq and differential expression analysis (DESeq2) were performed between the cytokine stimulated and the control keratinocytes or fibroblasts. Genes with adjusted p-value <0.05 that are also highly expressed in the cytokine stimulated keratinocytes were considered cytokine induced genes. The module scores for the upstream regulators were calculated on the target gene lists from the Ingenuity Pathway Analysis software.

Ligand receptor interaction analysis

CellphoneDB (v2.0.0) was applied for ligand receptor analysis [3]. The Seurat normalized counts and subcluster annotation for each cell were input into cellphoneDB to determine the potential ligand receptor pairs. Pairs with p value >0.05 were filtered out from further analysis. The subclusters were divided into NS specific subcluster (with NS composition > 70%), HS specific subcluster (with HS composition > 70%) and mixed subcluster. CellphoneDB was then run on the NS cells from the NS specific subclusters and HS cells from the HS specific subclusters. The pairs from the subclusters for the same cell type were merged to find ligand-receptor pairs between the major cell types. The pairs with higher interaction score in HS were plotted. The connectome web was plotted using the R package igraph.

Pseudotime trajectory construction

Pseudotime trajectory was constructed using the R package Monocle [4]. The raw counts for cells were extracted from the Seurat analysis and normalized by the estimateSizeFactors and estimateDispersions functions with the default parameters. Genes with average expression larger than 0.5 and detected in more than 10 cells were retained for further analysis. Variable genes were determined by the differentialGeneTest function with a model against the Seurat subcluster identities for the subtype identities of the NS or HS cell types. The orders of the cells were determined by the orderCells function, and the trajectory was constructed by the reduceDimension function with default parameters. The heatmap showing specific genes along pseudotime was plotted using the plot_pseudotime_heatmap function. Differential expression between pseudo-time states was carried out using the Seurat function FindMarkers. Ingenuity Pathway Analysis was used to determine the upstream regulators for the DEGs.

Spatial transcriptomics library preparation

Four lesional HS skin samples were frozen in OCT medium and stored at -80°C until sectioning. Optimization of tissue permeabilization was performed on 20-μm-thick sections using Visium Spatial Tissue Optimization Reagents Kit (10X Genomics, Pleasanton, CA, USA), which established an optimal permeabilization time to be 24 minutes. Samples were mounted onto a Gene Expression slide (10X Genomics), fixed in ice-cold methanol, stained with hematoxylin and eosin, and scanned under a microscope (Keyence, Itasca, IL, USA). Tissue permeabilization was performed to release the poly-A mRNA for capture by the poly(dT) primers that are precoated on the slide and include an Illumina TruSeq Read, spatial barcode, and unique molecular identifier (UMI). Visium Spatial Gene Expression Reagent Kit (10X Genomics) was used for reverse

transcription to produce spatially barcoded full-length cDNA and for second strand synthesis followed by denaturation to allow a transfer of the cDNA from the slide into a tube for amplification and library construction. Visium Spatial Single Cell 3′ Gene Expression libraries consisting of Illumina paired-end sequences flanked with P5/P7 were constructed after enzymatic fragmentation, size selection, end repair, A-tailing, adaptor ligation, and PCR. Dual Index Kit TT Set A (10X Genomics) was used to add unique i7 and i5 sample indexes and generate TruSeq Read 1 for sequencing the spatial barcode and UMI and TruSeq Read 2 for sequencing the cDNA insert, respectively. Libraries were sequenced on the Illumina NovaSeq 6000 sequencer to generate 150 bp paired end reads.

Spatial transcriptomics data analysis

After sequencing, the reads were aligned to the human genome (hg38), and the expression matrix was extracted using the spaceranger pipeline. Seurat was then used to analyze the expression matrix. Specifically, the SCTransform function was used to scale the data and find variable genes with default parameters. PCA and UMAP were applied for dimensional reduction. The FindTransferAnchors function was used to find a set of anchors between the scRNA-seq data and spatial-seq data, which were then transferred from the scRNA-seq to the spatial-seq data using the TransferData function. The major cell types obtained in the scRNA-seq data were used to annotate the spatial-seq data. The predicted cell type composition for each spot was then used to cluster the spots by the k-means algorithm. The clusters were annotated based on the average cell type prediction score across all the spots in the cluster.

Immunohistochemistry and trichrome staining

Formalin-fixed embedded human tissues were sectioned and heated at 65 ºC for 5 minutes, deparaffinized, and rehydrated. Slides were placed in pH9 antigen retrieval buffer and heated at 125 ºC for 30 seconds in a pressure cooker water bath. After cooling, slides were treated with 3% H_2O_2 (5 minutes) and blocked using 10% goat serum or horse serum (30 minutes). Overnight incubation (4°C) was then performed using anti-human primary antibody. Antibodies used were: CD3 (UM500048, 1:300), CD20 (AB9475, 1:20), CD11c (ab52632, 2µg/ml), CD31 (ab28364, 1:25), CD138 (LS-B9360, 1:40), KRT16 (LS-B7609, 2µg/ml), NE (MAB91671, 10µg/ml), ACTA2 (ab5694, 1:100), CD207 (PA5-82422, 1:200), CLEC9A (55451-I-AP, 1:70), LAMP3 (PA5-84069, 1:50), CD303 (Hpa029432, 1:20), CLEC10A (TA810180, 1:150), CD163 (MA5-11458, 1:25), LSP1 (LS-B16949-50, 2µg/ml) SFRP2 (LS-C794043, 1:300), SFRP4 (LS-C408100, 1:250), RAMP1 (AB203282, 1:200), COL11A1 (PA5-68410, 1:300), and CXCL13 (LS-C490370, 2µg/ml), YAP1 (ab205270, 1:2000), TAZ (HPA007415, 1:100), TEAD1 (#12292,1:100), TEAD2 (ab196669, 1:50), TEAD4 (H00007004, 3ug/ml). Slides were then washed, treated with secondary antibody (30 minutes), peroxidase (30 minutes) and diaminobenzidine substrate. Counterstain with Hematoxylin and dehydration was performed, and slides were mounted and viewed under the microscope. In addition, slides were stained with Masson's trichrome stain as previously described.

Immunofluorescence staining

Antigen retrieval was performed as described above. Formalin-fixed embedded human tissues were sectioned and heated at 65 ºC for 30 minutes, deparaffinized, and rehydrated. Slides were subsequently blocked and incubated with primary rabbit and mouse anti-human antibodies.

Primary rabbit antibodies were used ACTA2 (ab5694, 1:100), CD31 (ab28364, 1:25), LYVE1 (ab33682, 5μg/ml), CXCL13 (PA5-47035, 5μg/ml). Primary mouse antibodies used: CD31 (ab9498, 10μg/ml), HLA-DR (ab20181, 2μg/ml), CD3 (UM500048, 1:150), vimentin (AB92547, 1:200). Appropriate antibodies were co-incubated overnight at 4°C. Appropriate isotype control antibodies: rabbit IgG (ab172730), mouse IgG1 (ab 280974) both from Abcam, IgG2ak (14-4724-82) from Invitrogen, were stained in parallel with each set of the slides mentioned above. Slides were then washed three times for 5 min each with phosphate-buffered saline/ Tween 20 (PBST). All slides were then incubated with secondary antibodies fluorochrome-conjugated Alexa Fluor 594 conjugated anti-rabbit IgG (711-585-152) and Alexa Fluor 488 conjugated anti-mouse IgG (715-545-151) from Jackson Immuno Research. After 30 minutes coincubation, slides were washed three times for 5 min each with PBST. Mounted in an Prolong Dimond antifade with DAPI (Invitrogen). Photomicrographs were taken on Zeiss fluorescence microscope.

REFERENCS

1. Butler, A., Hoffman, P., Smibert, P., Papalexi, E. & Satija, R. Integrating single-cell transcriptomic data across different conditions, technologies, and species. *Nat Biotechnol* **36**, 411–420 (2018).
2. Billi, A. C. *et al.* Nonlesional lupus skin contributes to inflammatory education of myeloid cells and primes for cutaneous inflammation. *Sci Transl Med* **14**, eabn2263 (2022).
3. Efremova, M., Vento-Tormo, M., Teichmann, S. A. & Vento-Tormo, R. CellPhoneDB: inferring cell-cell communication from combined expression of multi-subunit ligand-receptor complexes. *Nat Protoc* **15**, 1484–1506 (2020).

4. Trapnell, C. *et al.* The dynamics and regulators of cell fate decisions are revealed by pseudotemporal ordering of single cells. *Nat Biotechnol* **32**, 381–386 (2014).

SUPPLEMENTAL TABLES

Supplemental Table 1. Patient characteristics

	HS patients n=5	
**Sex**		
Female, *n (%)*	3	(60)
Male, *n (%)*	2	(40)
**Age in years**, *mean ±SD*	42.22	±14.24
**BMI**, *mean ±SD*	28.95	±5.34
**Disease duration in years**, *mean ±SD*	20.00	±9.00
**Current or ex-smokers**, *n (%)*	5	(100)
**Hurley stage**		
Hurley stage II, *n (%)*	1	(20)
Hurley stage III, *n (%)*	4	(80)
**Anatomical location sampled**		
Groin, *n (%)*	4	(80)
Buttock, *n (%)*	1	(20)

HS, hidradenitis suppurativa; SD, standard deviation; BMI, body mass index

SUPPLEMENTAL FIGURES

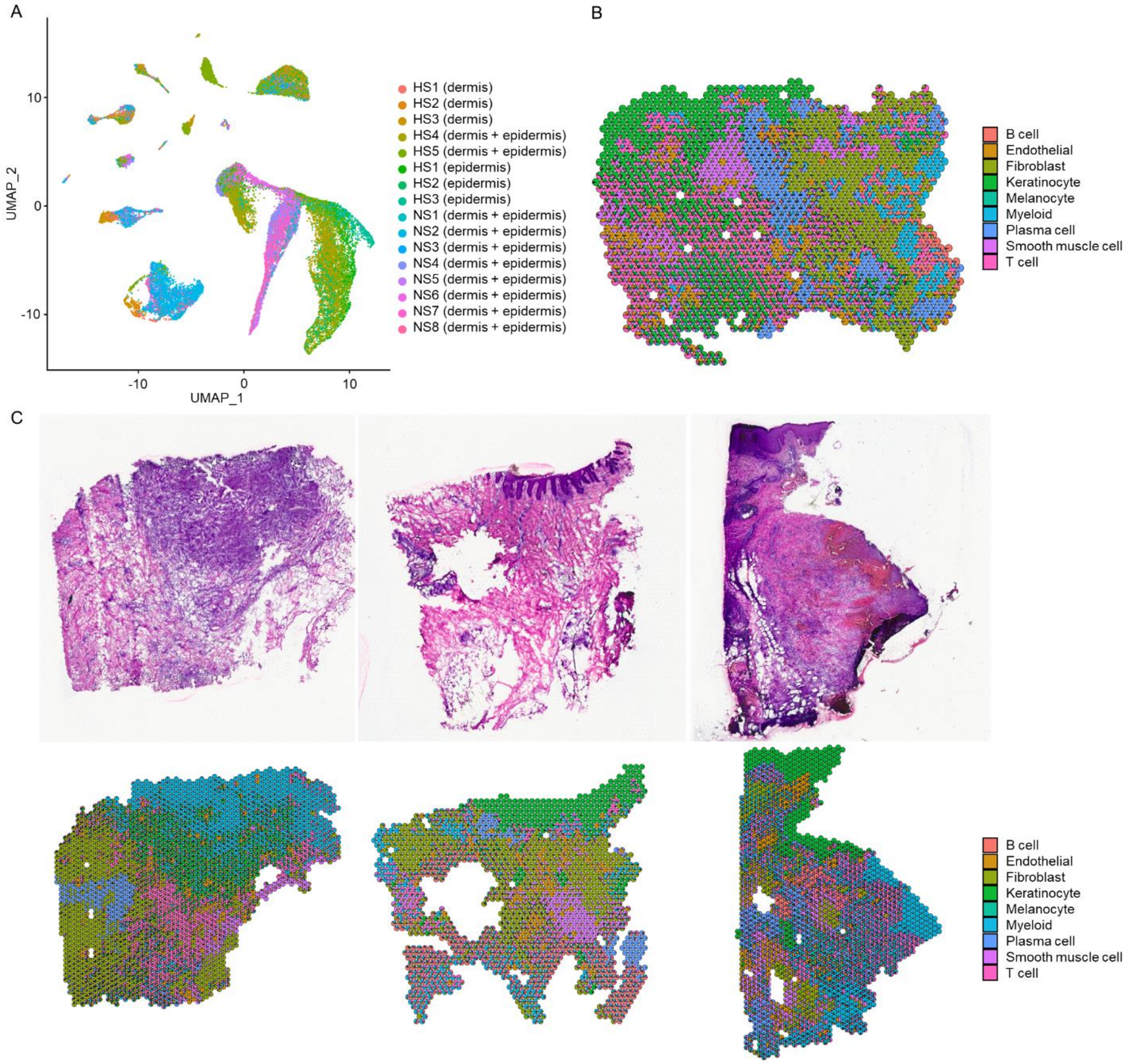

Figure S1. Cell composition and spatial transcriptomics of cell types in three more HS samples. **(A)** UMAP plot showing the cells colored by sequencing libraries. **(B)** Scatter pie plot showing the cell type composition of the HS spatial-seq sample. Each spot is represented as a pie chart showing the relative proportion of the cell types. **(C)** Spatial transcriptomics for the other three other HS samples. Top panel: H & E staining of the biopsy used for spatial transcriptomics; bottom panel: scatter pie plot showing the cell type composition.

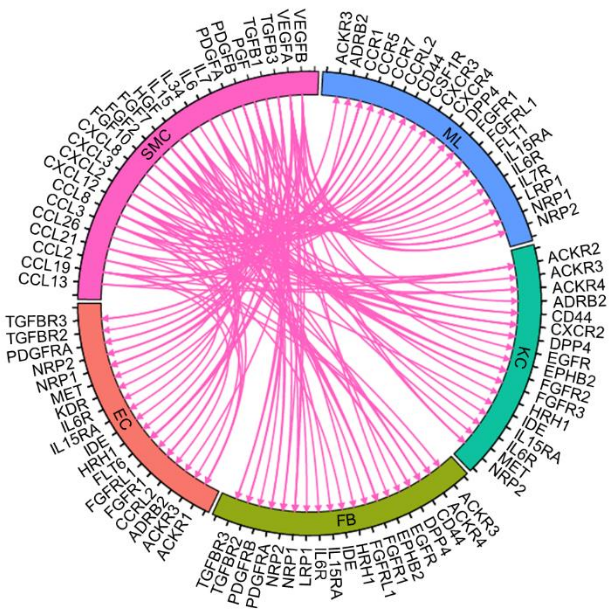

Figure S2. Ligand-receptor analysis of smooth muscle cells. Circos plot showing cytokine and growth factor ligand-receptor interactions with higher scores in HS compared with NS in which ligands are expressed by the smooth muscle cells (SMC) with receptors expressed by other cell types. ML, myeloid cells; FB, fibroblasts; EC, endothelial cells; KC, keratinocytes.

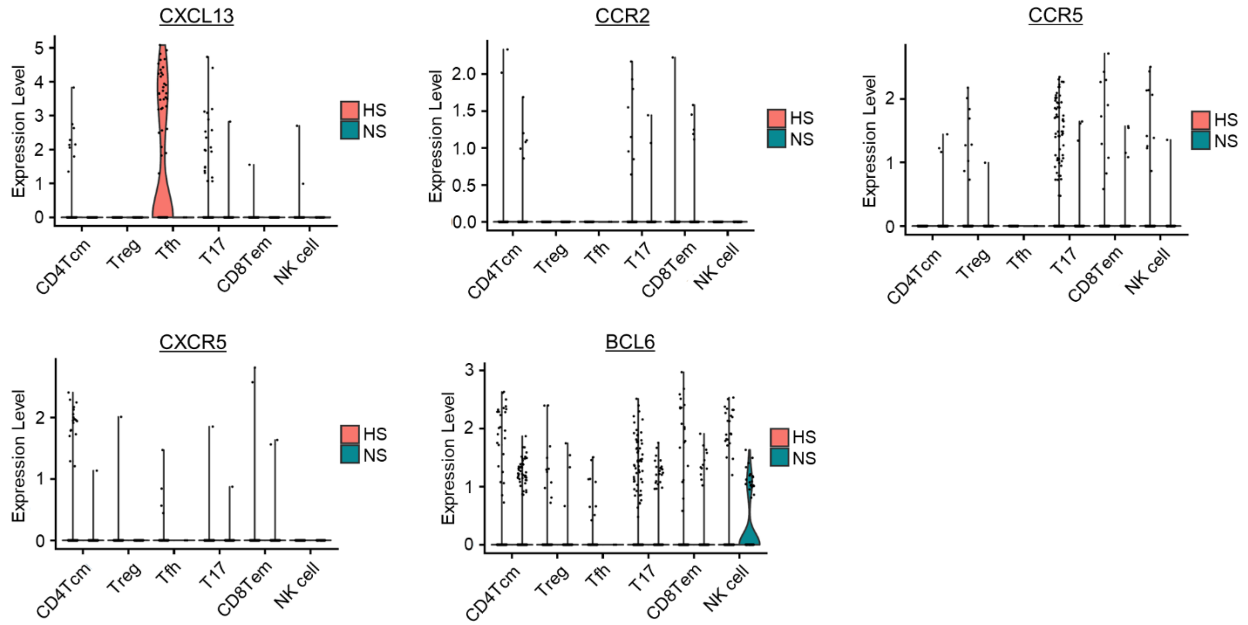

Figure S3. Expression of Tfh and Tph lineage makers among T cells.
Violin plots showing the expression of genes split by T cell subcluster. Each dot represents the gene's expression in a single T cell.

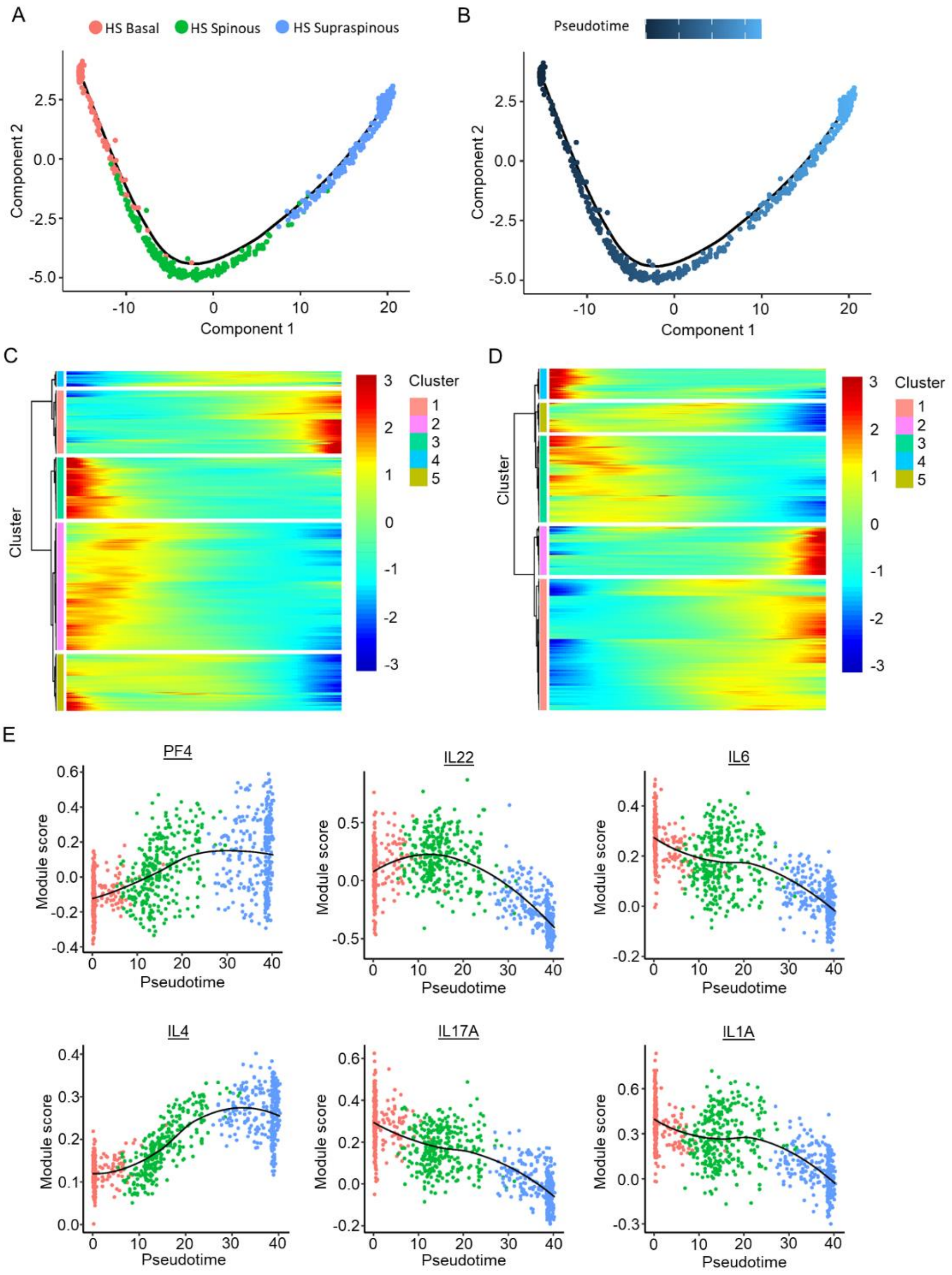

Figure S4. Pseudotime trajectory for NS keratinocytes.

(A) Pseudotime trajectory colored by the subtype identity of NS keratinocytes. **(B)** Pseudotime trajectory colored by the pseudotime; dark blue representing early, light blue representing late. **(C)** Heatmap showing the five expression patterns of variable genes along the pseudotime of the NS keratinocytes **(D)** Scatter plot showing the correlation between HS-derives keratinocyte pseudotimes and module scores for PF4 and IL4. The color represents the pseudotime subtype identity of the cell.

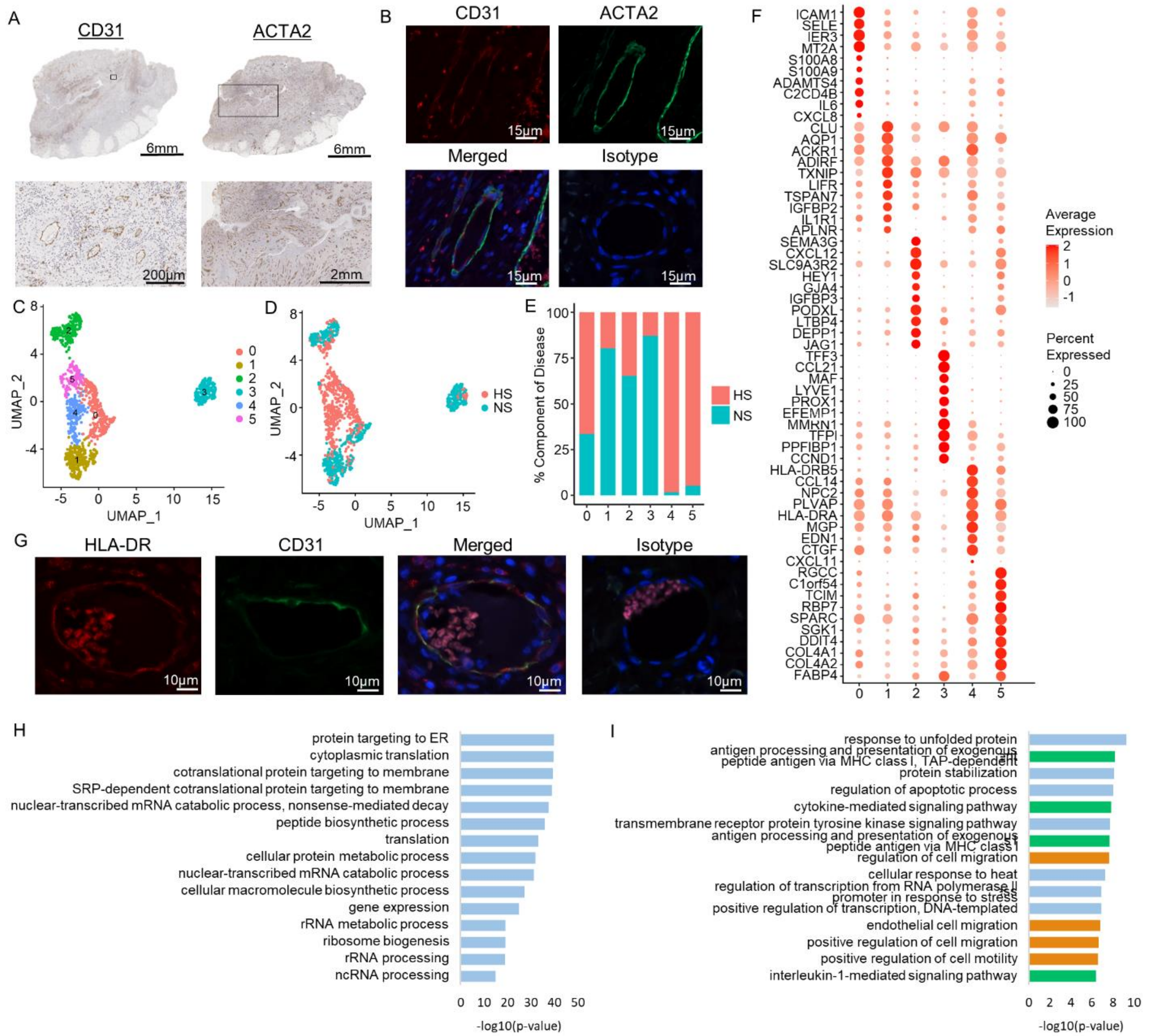

Figure S5. Immunologically active endothelial cell proliferation in HS lesional skin. Immunohistochemistry **(A)** and immunofluorescence **(B)** showing the prominent neovascularization in HS lesional skin. ACTA2; smooth muscle cells, CD31; endothelial cells. **(C)** UMAP plot showing 1,352 endothelial cells colored by subcluster. **(D)** UMAP plot showing the cells colored by disease conditions. HS: hidradenitis suppurativa; NS: normal/healthy skin. **(E)** Bar chart showing the cell clusters as percentage component of disease. **(F)** Dot plot showing the representative marker genes for each subcluster. The color scale represents the scaled expression of the gene. The size of the dot represents the percentage of cells expressing the gene of interest. **(I)** Representative immunofluorescence of HLA-DR and CD31 in HS lesional skin. **(H-I)** Bar chart showing enriched Gene Ontology Biological Processes in subcluster 4 **(H)** and 5 **(I)**, green; immune associated BP, orange; migration associated BP, blue; transcription/protein translation associated and other BPs.

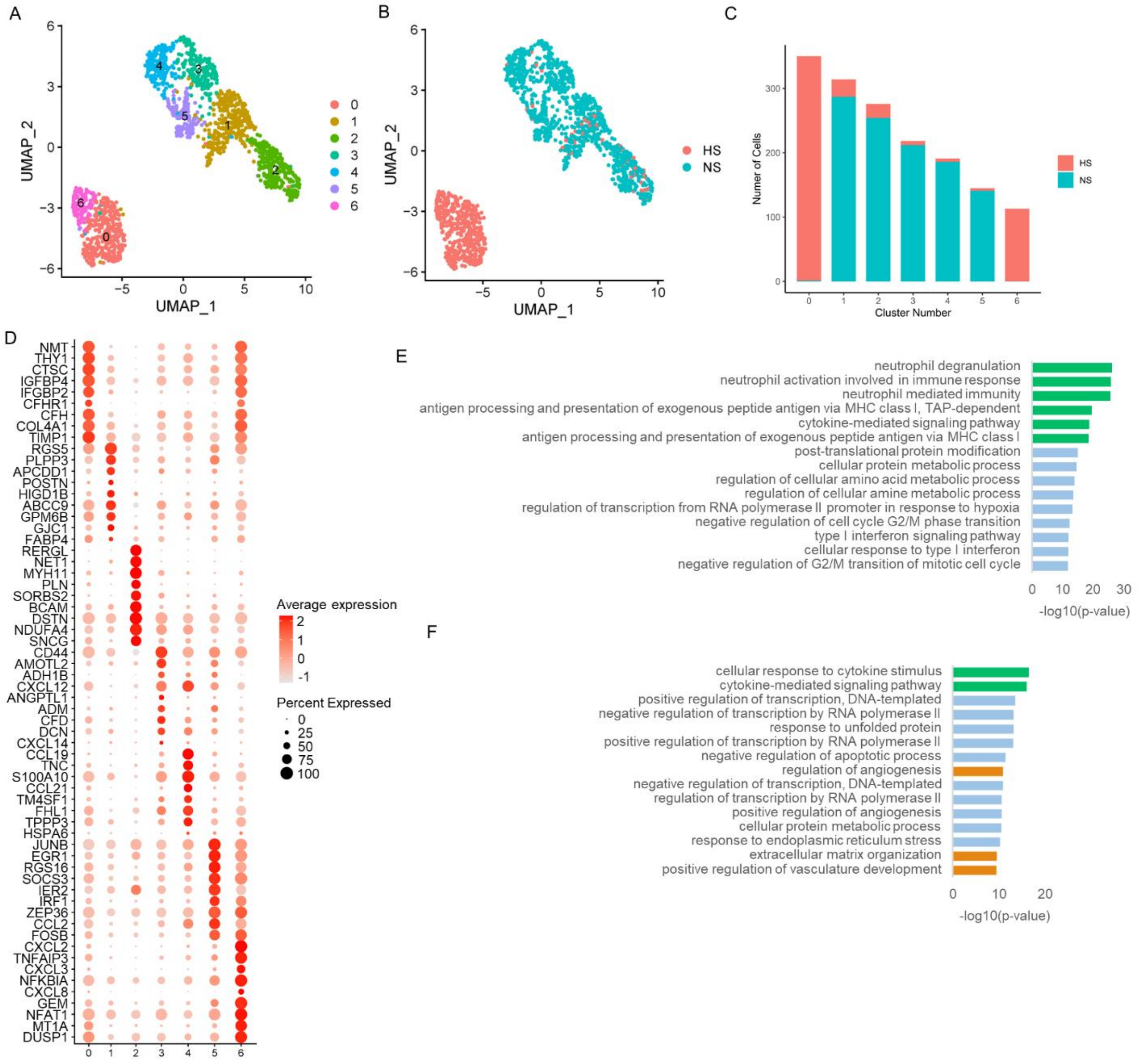

Figure S6. Immunologically active vascular smooth muscle cell proliferation in HS lesional skin. **(A)** UMAP plot showing 1,607 smooth muscle cells cells colored by subcluster. **(B)** UMAP plot showing the cells colored by disease conditions. HS: hidradenitis suppurativa; NS: normal/healthy skin. **(C)** Bar chart showing the cell clusters as percentage component of disease. **(D)** Dot plot showing the representative marker genes for each subcluster. The color scale represents the scaled expression of the gene. The size of the dot represents the percentage of cells expressing the gene of interest. **(E)** Bar chart showing enriched Gene Ontology Biological Processes in subcluster 0 and 6 **(F)**; green; immune associated BP, orange; angiogenesis associated BP, blue; transcription associated and other BPs.

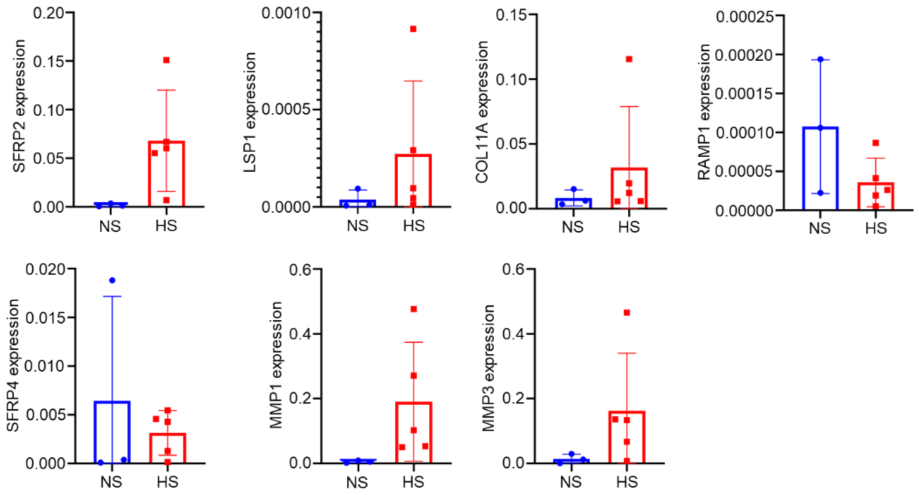

Figure S7. Expression patterns of HS primary fibroblasts.

Expression of fibroblast subtype marker genes among primary HS fibroblasts (n=5) and healthy control fibroblasts (n=3), normalized over *ACTB*; mean ± SD; unpaired T test/Mann-Whitney U test.

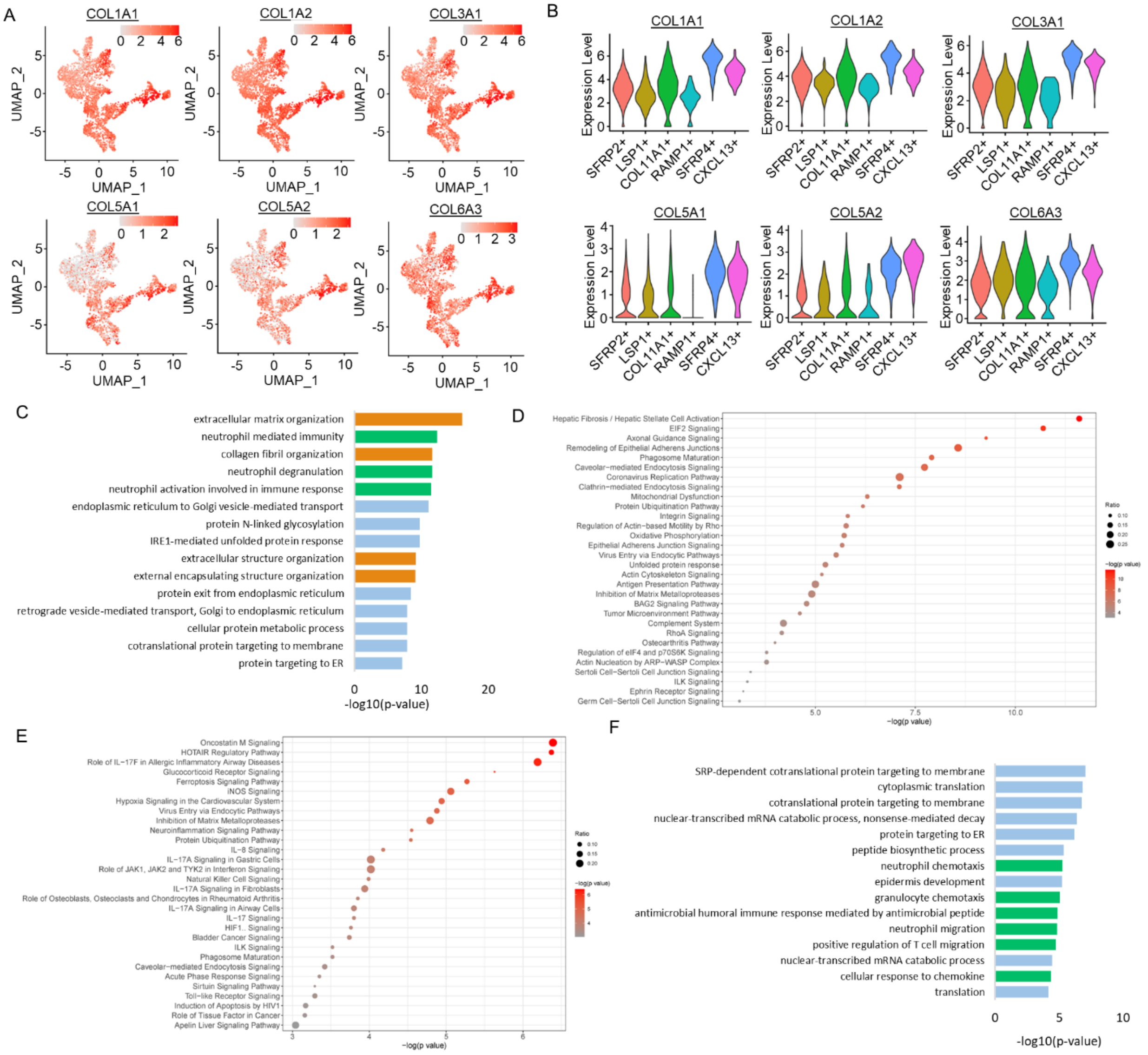

Figure S8. Biological functions and activation of *SFRP4*⁺ and *CXCL13*⁺ fibroblasts.
(A) UMAP plots and violin plots **(B)** showing the expression level of different collagen genes in fibroblasts. **(C)** Bar chart showing the top 15 enriched biological processes among *SFRP4*⁺ fibroblasts; green; immune associated BP, orange; extracellular matrix associated BP, blue; transcription associated and other BPs. **(D)** Dot plot showing the top 30 canonical pathways enriched in *SFRP4*⁺ and CXCL13+ **(E)** fibroblasts. **(F)** Bar chart showing the top 15 enriched biological processes among *CXCL13*⁺ fibroblasts; green; immune associated BP, blue; transcription associated and other BPs.

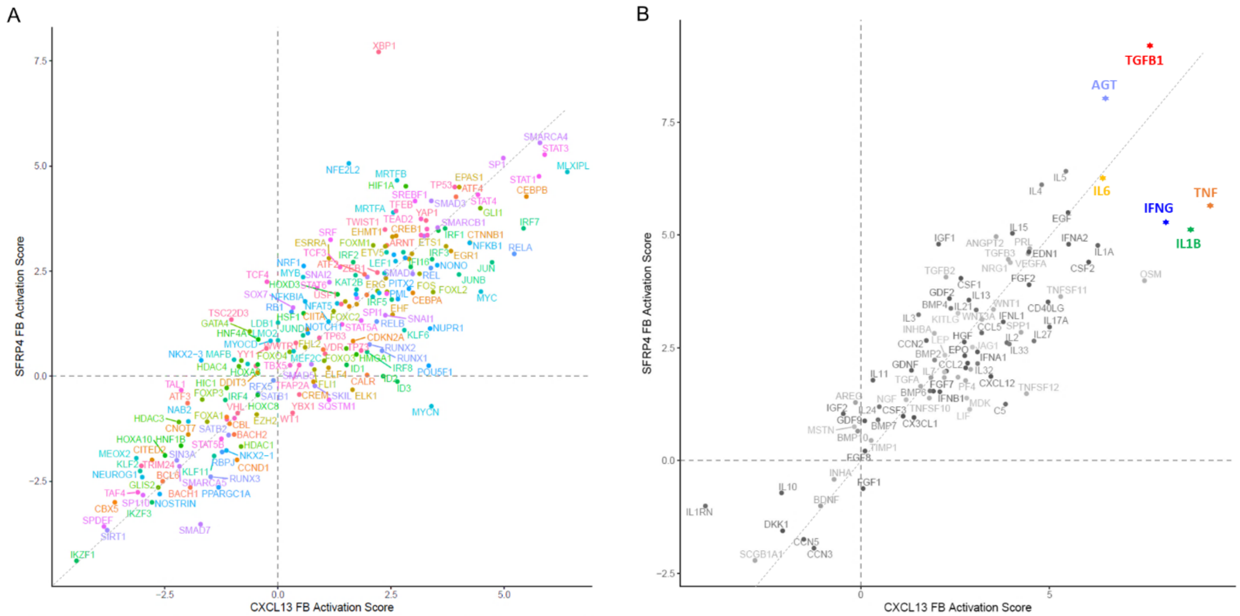

Figure S9. Activation of *SFRP4*⁺ and *CXCL13*⁺ fibroblasts.
(A) Scatter plot showing the activation z scores of activated transcription factor upstream regulators for the *SFRP4*$^{+}$ and *CXCL13*$^{+}$ FBs. **(B)** Scatter plot showing the activation z scores of activated cytokine and growth factor upstream regulators for the *SFRP4*$^{+}$ and *CXCL13*$^{+}$ FBs.

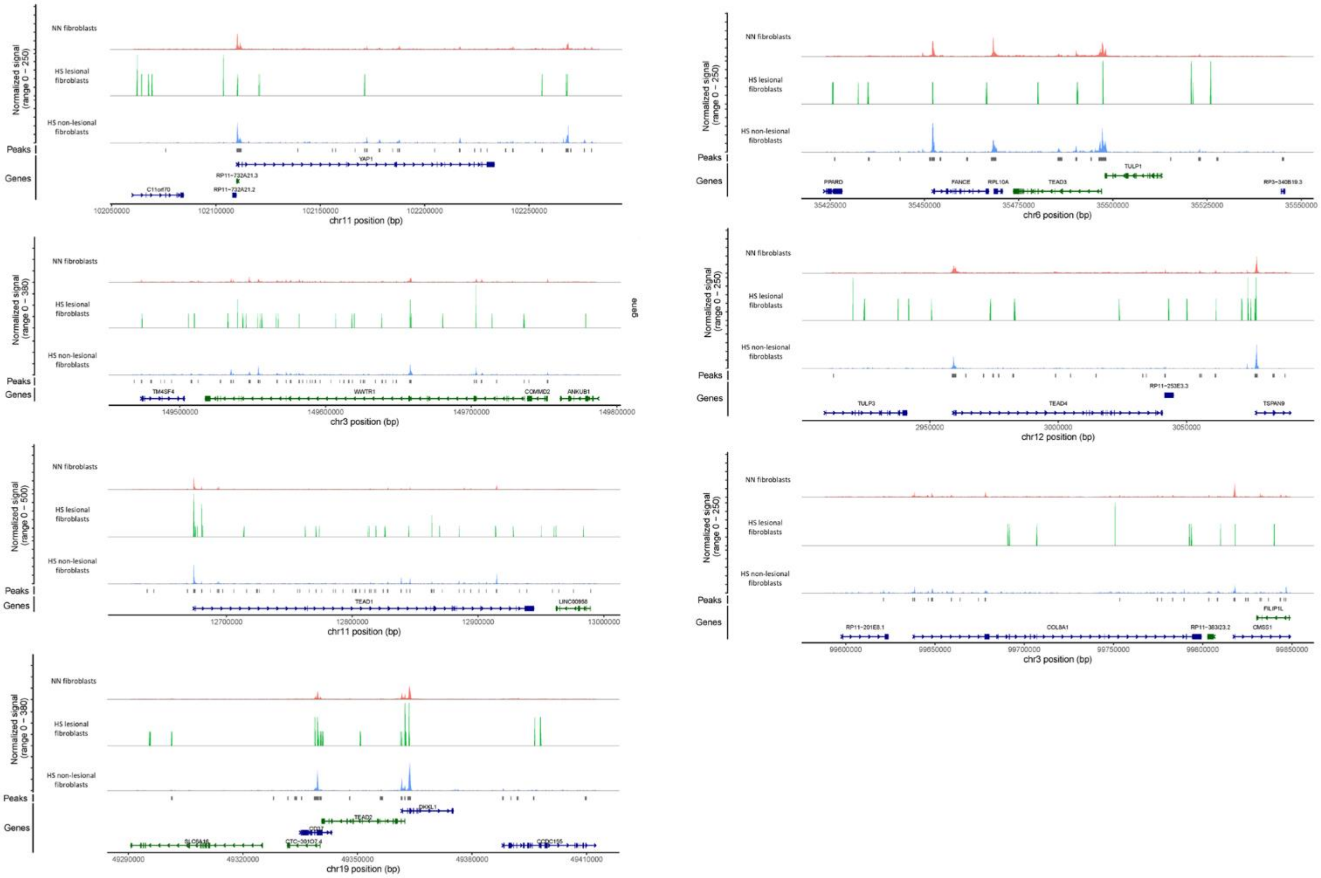

Figure S10. ATAC-seq of healthy and HS fibroblasts.

Figure showing increased chromatin accessibility as seen in the signal tracks and peaks for YAP1, WWTR1, TEAD1-4, and COL8A1 regions among HS lesional fibroblasts compared with both healthy and non-lesional HS fibroblasts. Data was obtained by performing ATAC-seq (assay of transposase accessible chromatin sequencing) on nuclei suspensions of chronic lesional and non-lesional HS skin samples of 5 patients and healthy skin from 5 controls. After quality check and alignment, peak calling was performed for YAP1, WWTR1, TEAD1-4, and COL8A1 using xxx to identify the chromatin accessibility of these regions.

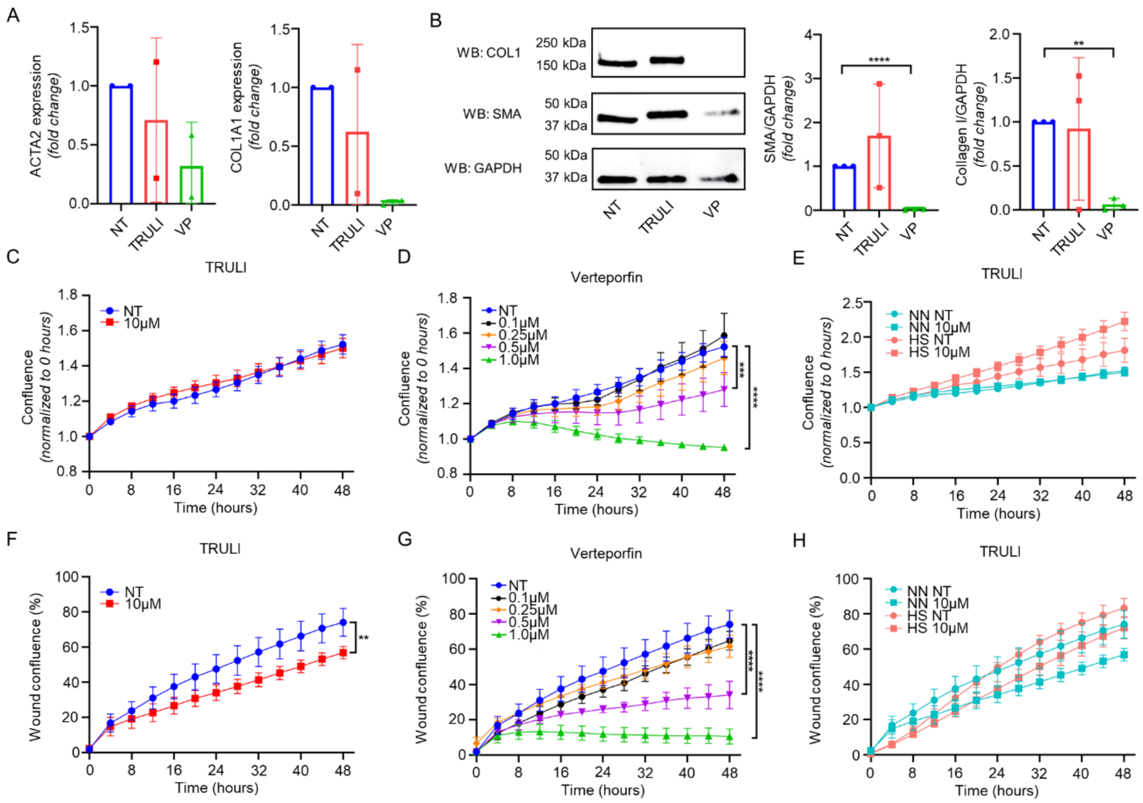

Figure S11. Modulation of the Hippo pathway in primary healthy fibroblasts.
(A) Quantitative PCR results showing the effect of TRULI (T10) or verteporfin (V10, both 10 μM) on ACTA2 and COL1A1 in healthy fibroblasts. N=2. **(B)** Effect of TRULI or verteporfin (both 10 μM) on SMA and COL1 protein levels in healthy fibroblasts by western blotting. Data normalized to NT. n=3; **p<0.01; **** p<0.0001, mean ± SD; . **(C)** TRULI showed not significant increase in cell proliferation while verteporfin **(D)** dose-dependently blocked cell growth among healthy fibroblasts (n=2, ****p<0.0001; mean ± SEM, two-way repeated measures ANOVA). Same NT groups shown in panels C/D. **(E)** HS fibroblast show greater proliferation potential compared with fibroblasts, which can be further induced by treatment with TRULI. **(F)** treatment with TRULI did not increase healthy fibroblast migration, where verteporfin **(G)** showed a dose-dependent reduction in cell migration (n=2, **p<0.01, ****p<0.0001; mean ± SEM, two-way repeated measures ANOVA). Same NT groups shown in panels F/G. **(H)** HS fibroblasts show greater cell migration at 48 hours compared with healthy fibroblasts, however TRULI does not further increase migration of either HS or healthy cells.
